# Betaine inhibits the stem cell-like properties of hepatocellular carcinoma by activating autophagy via SAM/m^6^A/YTHDF1-mediated enhancement on ATG3 stability

**DOI:** 10.7150/thno.102682

**Published:** 2025-01-06

**Authors:** Chen Wang, Meng-chu Li, Wen-ge Huang, Si-yu Huang, Maierhaba Wusiman, Zhao-yan Liu, Hui-lian Zhu

**Affiliations:** 1 Department of Nutrition, School of Public Health, Sun Yat-sen University, Guangzhou, 510080, China.; 2 Guangdong Provincial Key Laboratory of Food, Nutrition and Health, School of Public Health, Sun Yat-sen University, Guangzhou, 510080, China.; 3 Center of Experimental Animals, Sun Yat-sen University, Guangzhou, 510080, China.

**Keywords:** betaine, N^6^-methyladenosine, hepatocellular carcinoma, stem cell-like properties, autophagy

## Abstract

**Background:** Stem cell-like properties are known to promote the recurrence and metastasis of hepatocellular carcinoma (HCC), contributing to a poor prognosis for HCC patients. Betaine, an important phytochemical and a methyl-donor related substance, has shown protective effects against liver diseases. However, its effect on HCC stem cell-like properties and the underlying mechanisms remains uninvestigated.

**Methods:** We measured the effects of betaine on the stem cell-like properties and malignant progression of HCC using patient-derived xenografts, cell-derived xenografts, tail vein-lung metastasis models, *in vitro* limiting dilution, tumor sphere formation, colony formation, and transwell assays. Mechanistic exploration was conducted using western blots, dot blots, methylated RNA immunoprecipitation-qPCR, RNA stability assays, RNA immunoprecipitation-qPCR, RNA pull-down, and gene mutation assays.

**Results:** A cohort study of HCC found that a higher serum concentration of betaine was associated with decreased levels of stemness-related markers. Furthermore, in HCC cells and xenograft mice, betaine suppressed the stem cell-like properties of HCC by activating autophagy. Mechanistically, betaine increased the m^6^A modification in HCC by producing S-adenosylmethionine (SAM) via betaine-homocysteine S-methyltransferase (BHMT). This increase in SAM subsequently triggered autophagy by enhancing the stability of autophagy-related protein 3 (ATG3) via YTHDF1 in an m^6^A-dependent manner, thereby inhibiting the stem cell-like properties of HCC cells.

**Conclusions:** These findings indicate that betaine inhibits the stem cell-like properties of HCC via the SAM/m^6^A/YTHDF1/ATG3 pathway. This study underscores the potential anti-tumor effects of betaine on HCC and offers novel therapeutic prospects for HCC patients.

## Introduction

Hepatocellular carcinoma (HCC) is the third leading cause of cancer-related mortality globally and imposes a substantial health burden 1, 2. The main contributors to this poor prognosis are tumor recurrence and metastasis 3, 4. Previous studies have revealed that, the stem cell-like properties endow HCC cells with self-renewal and metastatic capabilities 5, 6. Furthermore, HCC patients with higher stemness indexes consistently experience a shorter survival duration 7-9. Thus, investigating the mechanisms underlying these stem cell-like properties and developing novel therapeutic strategies may improve the prognosis for HCC patients.

Autophagy is a fundamental and conserved cellular process that is regulated by a series of autophagy-related genes (ATGs), and plays a crucial role in modulating the stem cell-like properties of cancer 10, 11. Recent studies have reported that activating autophagy with natural products can impede the stem cell-like properties of cancer cells, including capsaicin, curcumin, and resveratrol 12, 13. Betaine, an important phytochemical in sugar beet and spinach 14, carries significant protective roles against several diseases, including mental disorders 15, muscle loss 16, reproductive disorders 17, 18, and liver-associated diseases 19-23. Experimental investigations found that these beneficial effects are associated with several mechanisms, such as modulating microglial M1/M2 differentiation 15, stimulating METTL21C/p97/VCP-dependent autophagy in myoblast 16, and mitigating senescence and apoptosis in reproductive cells 17, 18. More importantly, recent studies demonstrated that the protective effects of betaine on liver diseases are associated with activated autophagy 24, 25. However, whether betaine-activated autophagy could inhibit the stem cell-like properties of HCC cells, and the mechanisms behind betaine's activation of autophagy remain unclear.

N^6^-methyladenosine (m^6^A) is an epi-transcriptomic modification on RNAs 26, 27 and is instrumental in the activation of autophagy 28-30. Mechanically, m^6^A "writers" transfer a methyl group from S-adenosylmethionine (SAM) to RNAs, whereas "erasers" remove it. The "readers" like YTHDFs, YTHDCs, IGF2BPs, and HNRNPs recognize m^6^A in RNAs and regulate RNA processes including maturation, splicing, stability, and translation 31-33. Notably, betaine serves as a crucial donor of methyl groups by generating SAM, and plays an important role in modulating m^6^A modification 14, 34, 35. Hence, we hypothesize that betaine can inhibit the stem cell-like properties of HCC cells by activating autophagy via SAM-dependent m^6^A modification on ATGs.

In the present study, we aimed to investigate the inhibitory effects of betaine on the stem cell-like properties of HCC and to elucidate the underlying mechanisms. Our findings may provide further insights into the antitumor effects of betaine, and expand the range of therapeutic options for cancer patients.

## Materials and Methods

### Serum and tissue collection

The serum and patient tissues utilized in this study were derived from the on-going "Guangdong Liver Cancer Cohort (GLCC)" (NCT03297255), which was established in 2013 36. The criteria for patient recruitment were as follows: 1) newly diagnosed within the past month, 2) no history of other cancers, 3) no previous anticancer treatment, and 4) availability of fasting blood samples and surgical HCC tissues.

### Serum betaine measurement

Serum betaine was measured by high-performance liquid chromatography with online electro-spray ionization tandem mass spectrometry, following the methods described previously 37.

### Tumor sphere formation

To evaluate the stem cell-like properties of HCC cells, a tumor sphere formation assay was undertaken. Briefly, around 400 cells were seeded into a 6-well ultra-low plate containing 4 mL of serum-free medium. This medium was primarily constituted of DMEM-F/12 medium, supplemented with 1% B27, 1% N2, 25 ng/mL EGF, and 25 ng/mL FGF. The cells were then incubated for a period of 14 days. Afterwards, the formed tumor spheres were visualized and imaged using an inverted microscope.

### *In vitro* limiting dilution assay

After being diluted, HCC cells were added into a 96-well ultra-low plate at the density of 2, 5, 10, 25, 50 cells/well (n = 18 wells) and incubated with serum-free medium for 14 days. Afterwards, the number of wells with sphere were counted. The ability of sphere formation was calculated using online ELDA software in accordance with the instructions.

### Measurement of autophagosomes

After HCC cells were treated with betaine for 24 h, cells were washed with ice-cold PBS and harvested. Then, cells were fixed with 2.5% glutaraldehyde for 24 h and stained with phosphotungstic acid. Finally, cellular autophagosomes were captured and counted using a transmission electron microscope (TEM).

### Assessment of autophagic flux

HCC cells were planted into a six-well plate to reach a 40% confluence. Then, Ad-mCherry-GFP-LC3 (Beyotime, Shanghai, China) was added into each well and incubated for 48 h. Subsequently, the cells were planted into a confocal dish and cultured for 24 h. Afterwards, the cells were subjected to betaine treatment for 24 h and the LC3 puncta was captured using a con-focal microscope. The number of cellular LC3 puncta were analyzed by ImageJ software.

### Dot blots assay

After extraction, total RNA was diluted to a final concentration of 25 ng/μL and denatured at 95 ℃ for 6 min. Then, different concentrations of denatured RNA (50, 100, 200 ng) were added to a nitrocellulose membrane and cross-linked under an ultraviolet lamp for 16 min. Subsequently, the membrane was washed with TBST and incubated with 5% skim milk for 30 min. The remaining procedures followed the Western blots (WB) assay. Methylene blue (MB) staining was used as a loading control.

### m^6^A RNA methylation quantification

The level of RNA containing m^6^A modification in HCC cells was detected using EpiQuik m^6^A methylation quantification kit (Epigentek, Farmingdale, USA). After total RNA was extracted and mRNA was purified, the level of m^6^A in 100 ng of mRNA was measured according to the instruction of manufacturer.

### Methylated RNA immunoprecipitation-qPCR

The level of m^6^A-modified ATG3 in HCC cells was detected using the Methylated RNA Immunoprecipitation-qPCR (MeRIP-qPCR) method. After total RNA was extracted and purified, the extraction of m6A-modified RNA was performed using MeRIP kit (BersinBio, Guangzhou, China) according to the manufacturer's guideline. Then, isolated RNA was subjected to qPCR assay to detect the level of m^6^A-modified ATG3.

### RNA stability assay

HCC cells were planted into a 6-well plate and subjected to betaine treatment for 24 h. Then, the medium was removed, fresh medium containing 5 μg/mL Actinomycin D (MCE, Shanghai, China) was added into each well. Cells were harvested after different time of culture (0, 3, 6 h). The remaining procedures followed the RNA isolation and qPCR assay.

### RNA immunoprecipitation-qPCR

The RNA immunoprecipitation-qPCR (RIP-qPCR) kit (BersinBio) was used to detect the interaction between ATG3 mRNA and YTHDF1 protein. Briefly, HCC cells were lysed by RIP lysis buffer at 4 ℃ for 30 min. Meanwhile, 7.5 μg of YTHDF1 primary antibody was pre-bound to Protein A/G magnetic beads in immunoprecipitation buffer for 1.5 h. Then the cell lysates were added and rotated at 4 ℃ overnight. Afterward, RNA was eluted with elution buffer and extracted with TransZOL reagent. Enrichment of ATG3 mRNA was detected by qPCR assay. The IgG antibody was used as a negative control.

### RNA pull-down assay

RNA pull-down kit (BersinBio) was used to validate the interaction between ATG3 mRNA and YTHDF1 protein. The biotin-labelled ATG3 sense probe and the antisense probe were designed and synthesized by GenePharm. Briefly, the biotin-labelled ATG3 probe was added to streptavidin magnetic beads and precoated for 60 min. Then, cell lysates were mixed with the pre-coated magnetic bead and incubated at 4 °C for 150 min. Finally, proteins were eluted and subjected to WB assay.

### Construction of wild type and mutant cell with m^6^A-recognition site of YTHDF1

To validate that the binding of YTHDF1 protein and ATG3 mRNA was m^6^A dependent, the YTHDF1 specific m^6^A-recognition site mutant lentivirus was constructed by GenePharm (YTHDF1-MUT), and the wild type of YTHDF1 (YTHDF1-WT) was used as control (Table S1). After construction, the following procedures follow the process described in supplementary method section regarding the construction of stable transfected cell line.

### Animal purchase and feeding

Four-week BALB/c nude and NCG mice (male) were purchased from the Laboratory Animal Center of Sun Yat-sen University and Gempharmatech (Jiangsu, China), respectively. Mice were kept in an individual ventilated cage at the specific pathogen free (SPF) animal facility. All the animal studies have received permission from the animal experiments ethics committee (No. 2019-020), and conducted according to the institutional guidelines for the care and use of animals. After one week of acclimation, the mice were randomly assigned to different groups to carry out the following animal assays.

### *In vivo* limiting dilution assay

To detect the stem cell-like properties of HCC cells, *in vivo* limiting dilution assay was performed. Briefly, HCC spheres with different treatments were dissociated using accutase and suspended in PBS at a final density of 2×10^5^, 2×10^6^, 2×10^7^ cells/mL, respectively. Then, different concentrations of HCC cells (100 μL) were subcutaneous injected into mice (n = 5). After 30 days, the mice were humanely euthanized and tumors were extracted. The ability of tumor formation was calculated using online ELDA software in accordance with the instructions.

### Tail vein-lung metastasis assay

To investigate the metastatic ability, tail vein-lung metastasis assay was conducted. Briefly, HCC cells were subjected to different treatments for the generation of sphere cells. Then, sphere cells in different groups were dissociated with accuatse and diluted in PBS to reach a final density of 5×10^7^ cells/mL. Subsequently, 100 μL of cell mixture was injected into the lateral tail vein of mice (n = 8). The survival time of each mouse was recorded. Euthanasia was performed 66 days post-injection, and the lung tissue samples were obtained. Survival analysis was carried out to compare the difference of the survival time between different groups.

### Establishment of patient derived xenograft model

Patient derived xenograft (PDX) model was established to detect the effect of betaine on the stem cell-like properties, autophagy, and m^6^A modification in HCC tissues. Briefly, after surgery, the fresh HCC tissues were preserved in ice-cold Hanks Balanced salt Solution containing 5% antibiotic-antimycotic and delivered to the animal laboratory on ice within 2 h. Then, the HCC tissues were sectioned into a 1 mm^3^ fragments, and transplanted into the NCG mice on the subcutaneous side. After 90 days of first generation (G1) growth, the HCC tumor tissues from G1 mice were extracted and transplanted into NCG mice for second generation (G2) growth. After a further 60 days of growth, the G2 tumor tissues were isolated for the following intervention experiment.

In short, NCG mice were subjected to normal and 3% betaine containing drinking water for 7 days before the third generation (G3) transplantation. After G3 transplantation, mice in the normal and 3% betaine drinking group were randomly divided into 4 groups: 1) Control group: received normal drinking water and intraperitoneally injected with 100 µL of water every four days; 2) HCQ (Hydroxychloroquine) group: intraperitoneally injected with 100 µL of 60 mg/kg HCQ every four days; 3) Betaine group: received drinking water containing 3% betaine, and intraperitoneally injected with 100 µL of water every four days; 4) Betaine + HCQ group: received drinking water containing 3% betaine, and intraperitoneally injected with 100 µL of 60 mg/kg HCQ every four days. Mice were continually fed for 60 days. Subsequently, the mice were humanely euthanized and the HCC tumor tissues from G3 mice were collected for following assays.

### Statistical analysis

All data are presented as mean ± SD. The difference between two groups was calculated by the Student's *t*-test using SPSS 22.0 software (IBM, Armonk, USA). Survival analysis was performed using the Log-rank test. All statistical graphs and figures were generated using GraphPad Prism 8.0 (San Diego, USA) and Adobe Illustrator CS6 (Adobe, San Jose, USA) software, respectively. All experiments were repeated at least 3 times. A P value less than 0.05 was regarded as statistically significant.

## Results

### Betaine inhibits the stem-cell like properties of HCC cells

**Figure 1:**
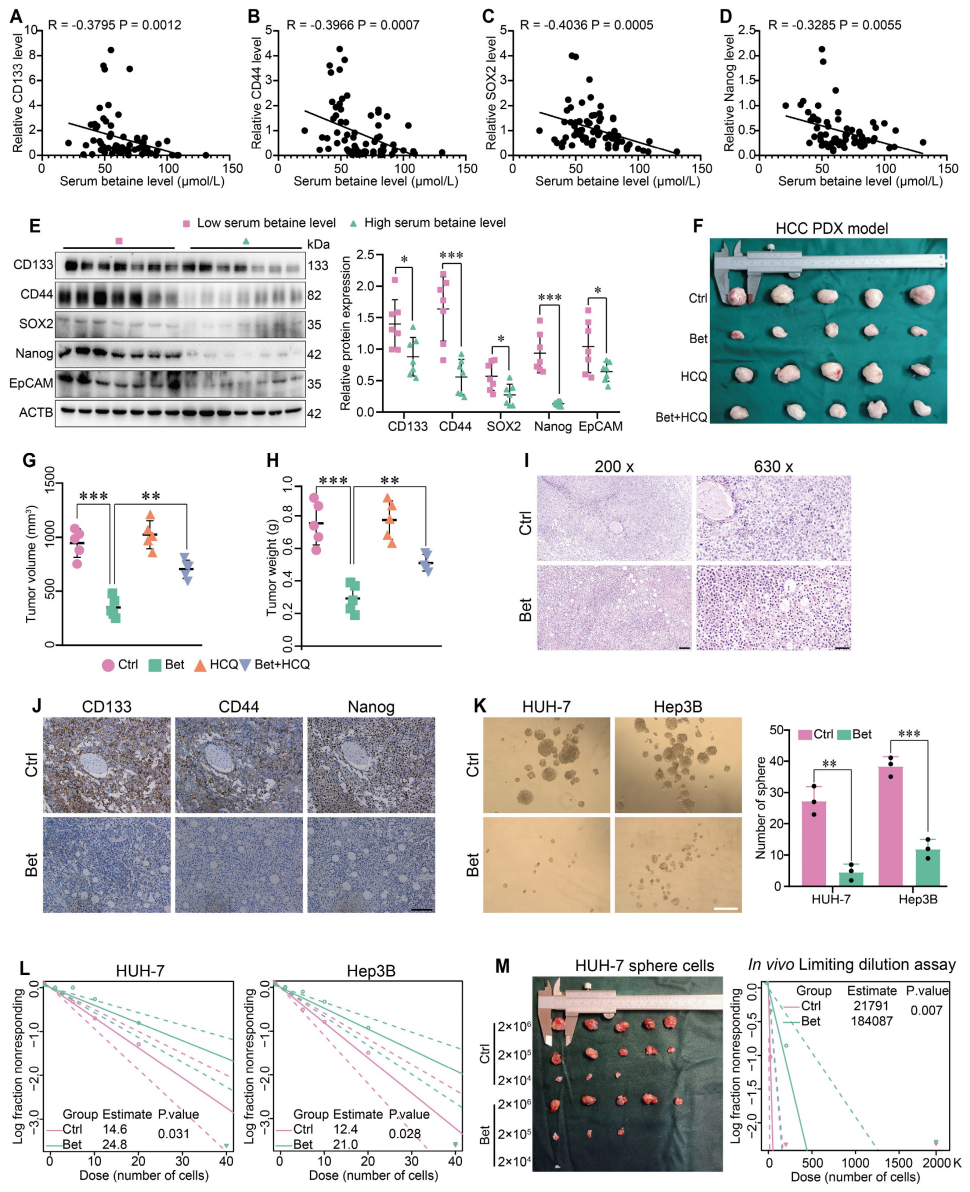
** Betaine inhibits the stemness of HCC cells.** (A-D) The correlation between serum betaine concentrations and the expression levels of stemness-related markers in HCC tissues from the GLCC cohort (n = 70) was analyzed using Pearson correlation coefficient. (E) HCC tissues were divided into four groups based on their serum betaine levels using the quartile method, seven HCC tissues were randomly chosen from the lowest quartile group to represent the low level of serum betaine group, and another seven HCC tissues were randomly selected from the highest quartile group to represent the high level of serum betaine group. The expression levels of CD133, CD44, SOX2, Nanog and EpCAM proteins were analyzed by WB assay. (F) Effects of betaine (Bet), HCQ, and Bet+HCQ on the growth of HCC were evaluated via an PDX model (n = 5/group). Tumor entity view. (G-H) Statistical analysis of tumor volume and tumor weight in PDX model among different groups. (I) HE staining of PDX tumor tissues (scale bars = 100 μm for left panel, and 40 μm for right panel). (J) IHC analysis of CD133, CD44, and Nanog in PDX tumor tissues with or without betaine treatment (scale bars = 100 μm). (K) Effects of betaine on the sphere formation ability of HCC cells were evaluated (scale bars = 100 μm). A statistical graph is shown. (L) Effects of betaine on the stemness of HCC cells were measured by *in vitro* limiting dilution assay (n = 18/group). (M) HCC cells were treated with betaine and subjected to sphere cells formation. Then, sphere cells were diluted in a gradient and injected subcutaneously into nude mice. The number of tumors in each group was counted after 30 days. The injected cell quantity and tumor formation frequency (n = 5 per group) were shown. *P < 0.05, **P < 0.01, ***P < 0.001.

To obtain a comprehensive understanding of betaine's impact on the stem cell-like properties of HCC, both the serum betaine levels and the expression of stemness-related markers in 70 HCC patients were assessed. The results indicated that patients with higher serum betaine levels exhibited reduced expression of stemness-related markers (Figure 1A-E and Table S2), suggesting that betaine may suppress the stem cell-like properties of HCC cells. Then, a PDX model of HCC was established and treated with betaine (Figure S1A). The findings revealed that betaine significantly inhibited tumor growth, as well as the volume and weight of HCC tumors (Figure 1F-H). Histologically, compared with the control (Ctrl) group, there was a reduction in nuclear atypia, mitotic figures, and the nuclear-to-cytoplasmic ratio after betaine (Bet) intervention. In addition, increased tumor cell pyknosis and dissolution of nuclei were observed (Figure 1I). This was accompanied by a downregulation of stemness-related markers in the betaine-treated group (Figure 1J and Figure S1B).

Subsequently, HCC cells were treated with 200 mM of betaine to investigate its impact on their stem cell-like properties (Figure S1C-F).* In vitro* assays demonstrated that betaine treatment significantly attenuated the stem cell-like properties of HCC cells, manifested by the downregulation of stemness-related markers, a decrease in sphere number, and a reduction in sphere formation capacity (Figure 1K-L and Figure S1G). Moreover, *in vivo* limiting dilution assay confirmed that betaine markedly suppressed the tumorigenic abilities of HCC sphere cells (Figure 1M). In summary, these results collectively demonstrate that betaine effectively inhibits the stem cell-like properties of HCC.

### Amelioration of the malignant progression of HCC following suppression of stem cell-like properties by betaine

**Figure 2:**
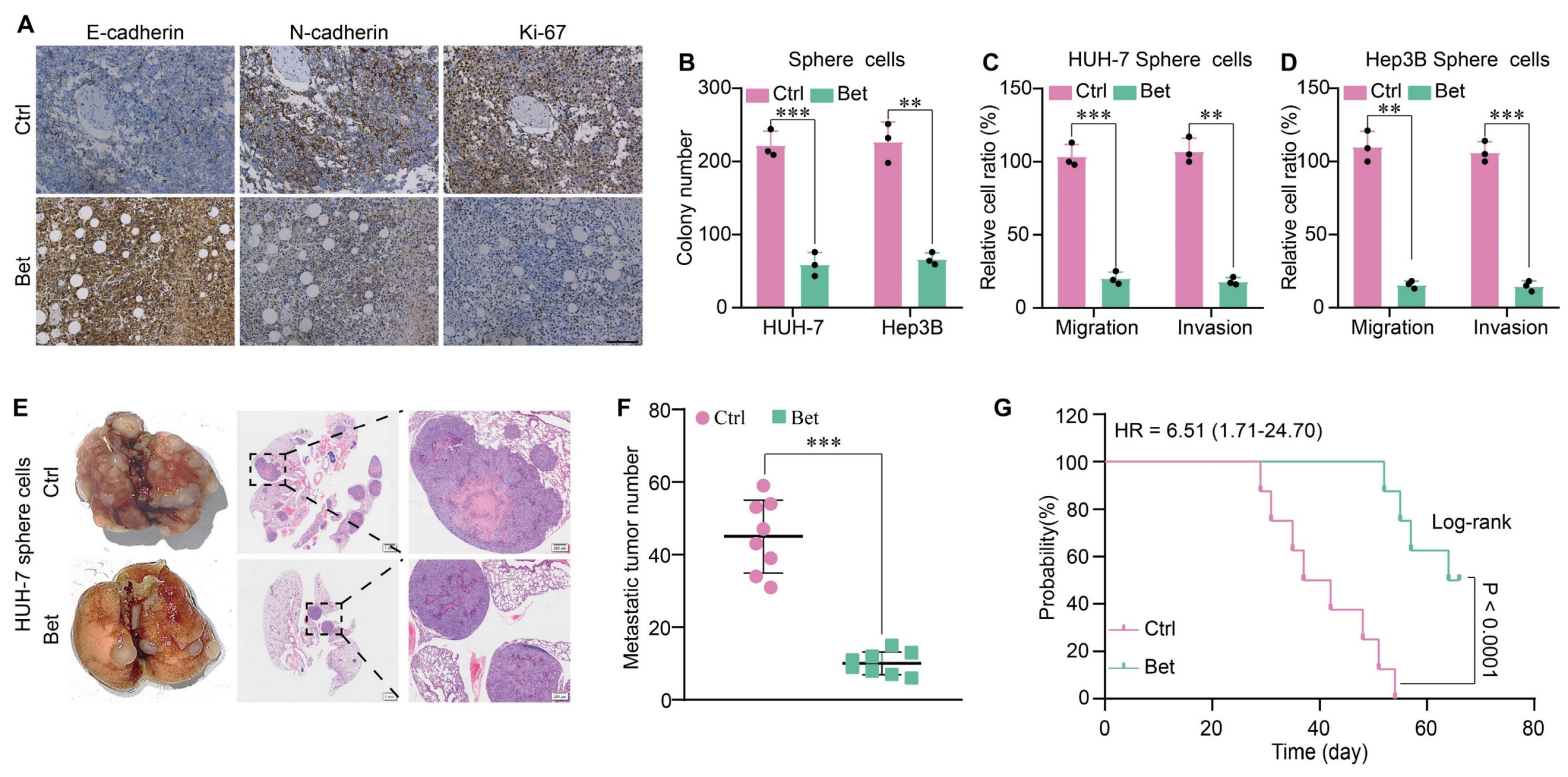
** Betaine inhibits the malignant progression of HCC.** (A) IHC analysis of E-cadherin, N-cadherin, and Ki-67 in PDX tumor tissues with or without betaine treatment (scale bars = 100 μm). (B-D) HCC cells were treated with betaine and subjected to sphere cells formation, then sphere cells were dissociated and subjected to colony formation and transwell assays to detect their clonogenic, migrative and invasive capabilities. Statistical graphs are shown. (E) HCC cells were treated with betaine and subjected to sphere cells formation, then sphere cells were dissociated and 5×10^6^ of cells were injected into the tail vein of nude mice. After 66 days of injection, mice were humanely euthanized. Images of lung metastatic nodules and H&E staining are shown (scale bar = 1 mm for left panel, and 200 μm for right panel). (F) Quantitative results of lung metastatic nodules for the Bet and Ctrl groups. (G) Kaplan-Meier survival curve analysis of nude mice injected with HCC sphere cells (n = 8/group). HR: Hazard Ratio. **P < 0.01, ***P < 0.001.

The stem cell-like properties of cancer cells are associated with malignant cellular behaviors, including increased growth, metastasis, and epithelial-mesenchymal transition (EMT). Therefore, we evaluated the change of growth and metastasis of HCC cells after the inhibition of stem cell-like properties by betaine. WB and IHC assays indicated that betaine notably suppressed the expression of the protein markers associated with growth and metastasis and inhibited EMT in PDX tissues (Figure 2A and Figure S2A). In addition, sphere cells derived from betaine-treated HCC cells showed decreased viability and motility, as evidenced by inhibited colony formation, migration, and invasion (Figure 2B-D and Figure S2B-C). Similarly, the expression of protein markers associated with growth, metastasis, and EMT was downregulated in betaine-treated sphere cells (Figure S2D). Lastly, in a mouse model of tail vein metastasis, mice injected with betaine-treated sphere cells exhibited decreased metastasis and extended survival times (Figure 2E-G and Figure S2E). Thus, our data demonstrate that betaine ameliorates the malignant progression of HCC.

### Betaine inhibits the stem cell-like properties of HCC by activating autophagy

**Figure 3:**
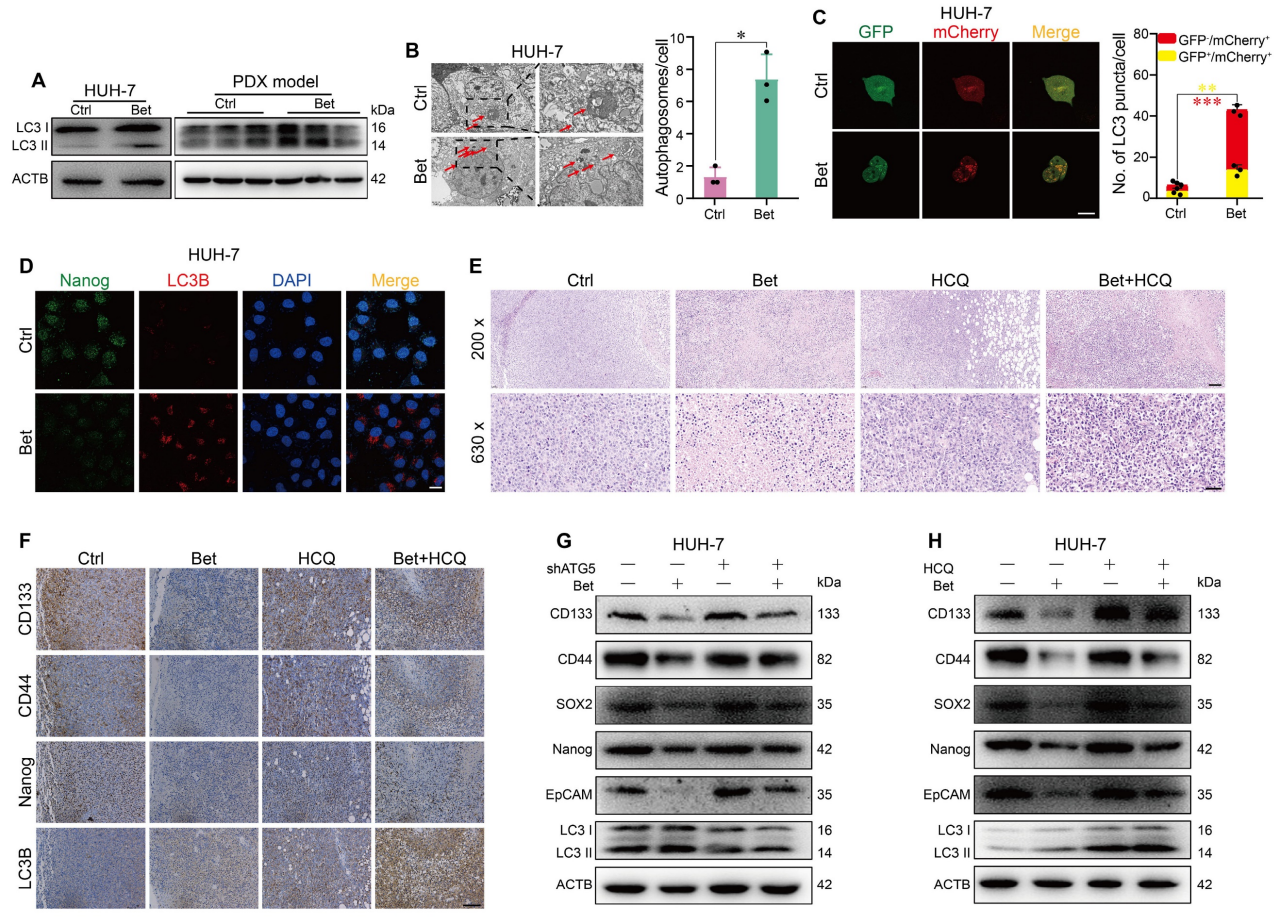
** Betaine inhibits the stem cell-like properties of HCC by activating autophagy.** (A) After betaine treatment, the expression levels of LC3 I/II protein in HUH-7 cells and PDX tumor tissues were analyzed by WB assay. (B) HUH-7 cells were treated with betaine, and the intracellular autophagosomes were captured via a TEM (scale bar = 2 μm for left panel and 1 μm for right panel). A statistical graph is shown. (C) HUH-7 cells were pre-transfected with mCherry-GFP-LC3B, and then subjected to betaine treatment. The red and yellow puncta were captured by a confocal microscope and quantitated (scale bar = 20 μm). A statistical graph is shown. (D) HUH-7 cells were treated with betaine, the expression levels of Nanog and LC3B proteins in the same cells were detected by IF assay (scale bar = 20 μm). (E) HE staining of PDX tumor tissues (scale bar = 100 μm for upper panel, 40 μm for lower panel). (F) After being exposed to betaine, HCQ, or combined betaine and HCQ treatments, IHC analysis of CD133, CD44, Nanog, and LC3B in PDX tumors (scale bars = 100 μm). (G) HUH-7 cells were pre-transfected with shATG5, and then subjected to betaine treatment. The expression levels of CD133, CD44, SOX2, Nanog, EpCAM, and LC3B proteins were analyzed by WB assay. (H) HUH-7 cells were subjected to betaine, HCQ or combined betaine and HCQ treatments, and the expression levels of CD133, CD44, SOX2, Nanog, EpCAM, and LC3B proteins were analyzed by WB assay. *P < 0.05, **P < 0.01, ***P < 0.001.

Previous study has indicated that the protective effect of betaine on liver disease is linked to increased autophagy 25. Therefore, we investigated the impact of betaine on autophagy in HCC cells. Our findings revealed that betaine treatment significantly elevated the levels of LC3 II in HCC cells and PDX tissues (Figure 3A, and Figure S3A-B). The observed increase in LC3 II levels could be due to either the activation of autophagy or the inhibition of autophagosome degradation. To clarify this, we examined the autophagic flux in HCC cells treated with betaine. TEM and mCherry-GFP-LC3 transfection results demonstrated that betaine treatment significantly promoted autophagosome formation and contributed to a higher red/yellow puncta ratio (Figure 3B-C and Figure S3C-D), suggesting the activation of autophagy by betaine in HCC cells.

To investigate whether the inhibitory effects of betaine on the stem cell-like properties of HCC cells are linked to the activation of autophagy, we first examined the change in stemness- and autophagy-related markers in HCC cells with betaine treatment. IF assay showed that the expression of Nanog was downregulated while LC3B was upregulated in HCC cells after betaine treatment (Figure 3D and Figure S3E), indicating the negative correlation of stem cell-like properties and autophagy in HCC cells under betaine treatment. Then, we utilized HCQ (a terminal autophagy inhibitor) to inhibit autophagy in PDX tumors and found that inhibition of autophagy impaired the antitumor effects of betaine on HCC (Figure 1F-H). Histologically, compared with the betaine group, there was an increase in nuclear atypia, mitotic figures, nuclear-to-cytoplasmic ratio, and reduced tumor cell pyknosis and dissolution of nuclei were observed in Bet+HCQ group (Figure 3E). In addition, a significant elevated stemness-related marker was detected in Bet+HCQ group compared to Bet group (Figure 3F and Figure S3F-H). Subsequently, autophagy was inhibited in HCC cells using HCQ and ATG5 shRNA/siRNA treatments, and the changes in stem cell-like properties were observed (Figure S4A-B, S4G-H, and S5A-H). Results revealed that autophagy inhibition significantly reduced the inhibitory effects of betaine on the stem cell-like properties of HCC cells, as demonstrated by upregulated stemness-related markers and sphere formation ability (Figure 3G-H and Figure S6A-D).

**Figure 4:**
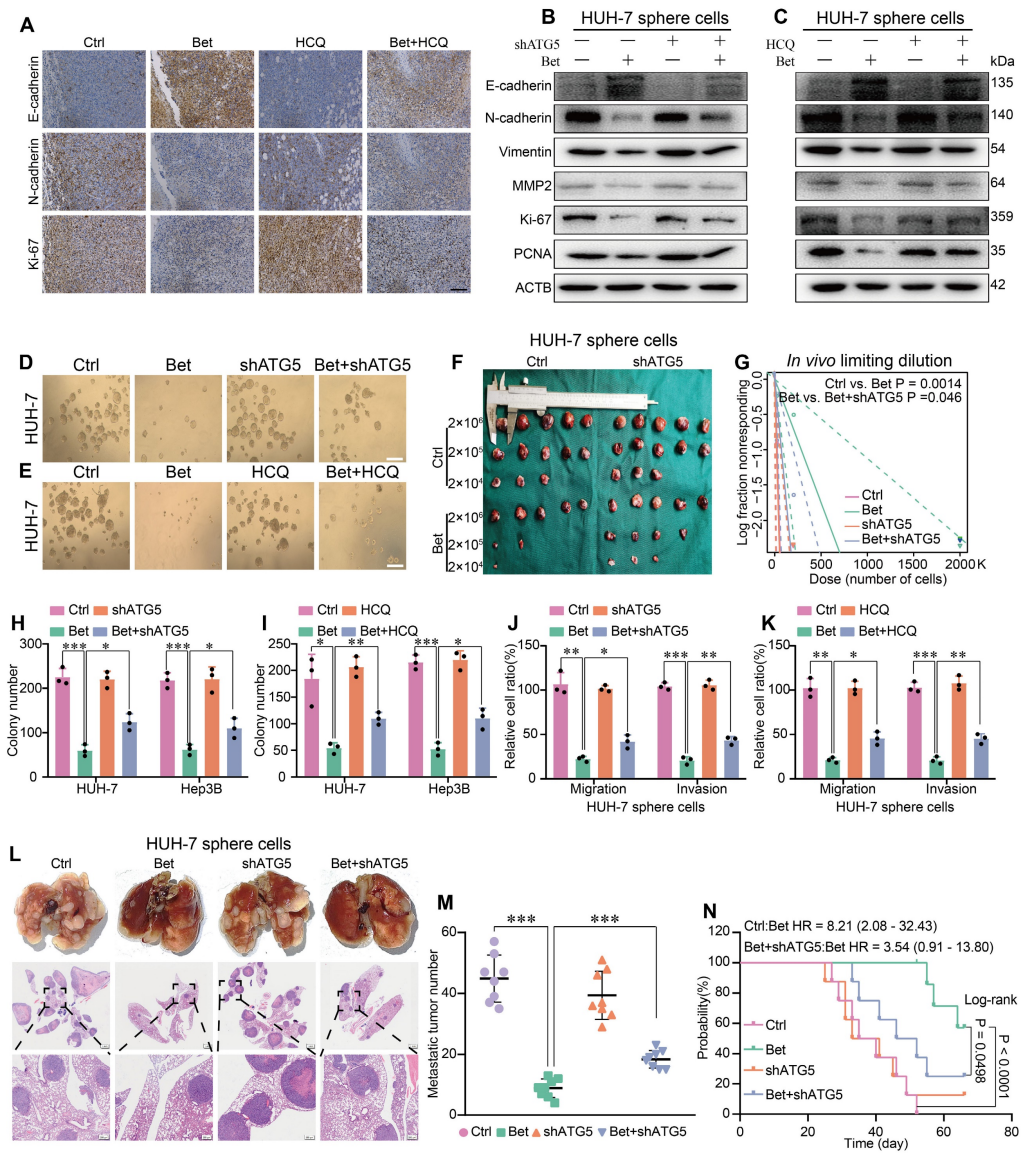
** Inhibition of autophagy impairs the antitumor effects of betaine on HCC.** (A) IHC results of E-cadherin, N-cadherin, and Ki-67 in PDX tumor tissues (scale bars = 100 μm). (B-C) WB analysis of the expression levels of E-cadherin, N-cadherin, Vimentin, MMP2, Ki-67, and PCNA proteins in HUH-7 sphere cells. (D) HUH-7 cells were pre-transfected with shATG5, and then were subjected to betaine treatment. The tumor sphere formation ability of HUH-7 cells under different treatments was measured (scale bars = 100 μm). (E) HUH-7 cells were subjected to betaine, HCQ, or combined betaine and HCQ treatments, and the tumor sphere formation ability of HUH-7 cells under different treatments was measured (scale bars = 100 μm). (F-G) HUH-7 cells were pre-transfected with shATG5 and subjected to betaine treatment for generation of sphere cells. Then, HUH-7 sphere cells were diluted in a gradient and injected subcutaneously into nude mice. The number of tumors in each group was counted after 30 days. The injected cell quantity and tumor formation frequency (n = 5 per group) were shown. (H-K) HUH-7 cells were pre-transfected with shATG5 or treated with HCQ, then subjected to betaine treatment for generation of sphere cells. After being dissociated and diluted, sphere cells were subjected to colony formation, and transwell migration and invasion assays. Statistical graphs are shown. (L) HUH-7 cells were pre-transfected with shATG5 and subjected to betaine treatment for generation of sphere cells. After being dissociated and diluted, 5×10^6^ of cells were injected into the tail vein of nude mice. After 66 days of injection, mice were humanely euthanized. Images of lung metastatic nodules and H&E staining were shown (scale bar = 1 mm for upper panel, and 200 μm for lower panel). (M) Quantitative results of lung metastatic nodules in different groups. (N) Kaplan-Meier survival curve analysis of nude mice injected with HCC cells in different groups (n = 8/group). HR: Hazard Ratio. *P < 0.05, **P < 0.01, ***P < 0.001.

Subsequently, we measured the change of malignant cellular phenotypes of HCC following upregulation of stemness. Results showed that the inhibition of EMT, metastasis, and growth-related markers by betaine treatment was reversed when autophagy was suppressed in PDX tumors and HCC cells (Figure 4A-C and Figure S7A-C). Meanwhile, sphere formation, colony formation, transwell, and* in vivo* tumor growth assays showed that both HCQ treatment and ATG5 silencing significantly mitigated the inhibitory effects of betaine on these cellular phenotypes (Figure 4D-K and Figure S7D-M). Furthermore, an *in vivo* metastasis assay showed that the inhibition of autophagy markedly increased the metastatic ability and shortened the survival time of mice compared to the Bet group (Figure 4L-N). Hence, our findings demonstrate that the inhibition of autophagy attenuates the effects of betaine on the stem cell-like properties and malignant phenotypes of HCC.

### Betaine promotes autophagy via SAM-mediated m^6^A modification

**Figure 5:**
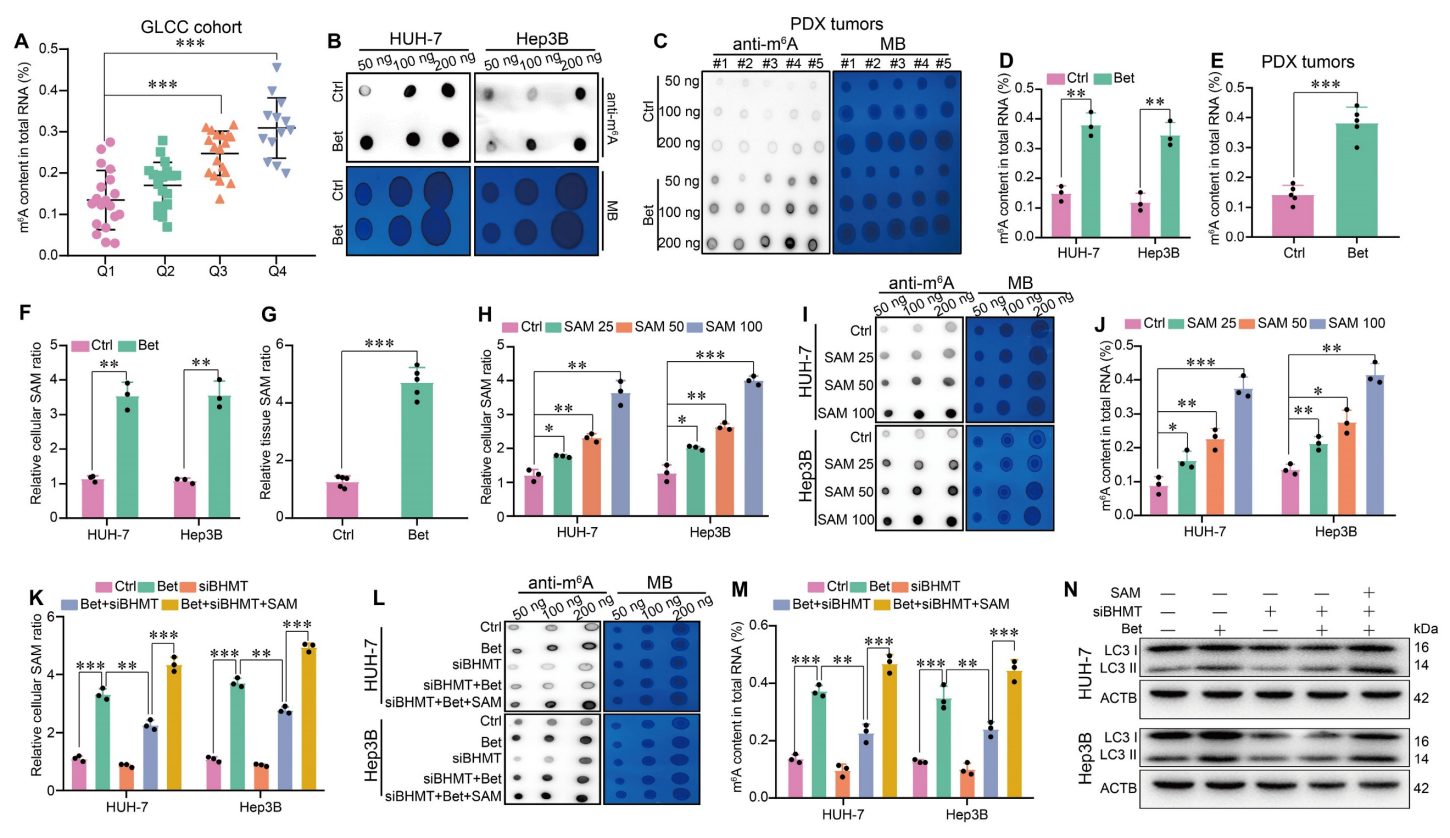
** Betaine activates autophagy via SAM-mediated m6A modification.** (A) HCC tissues from GLCC cohort (n = 70) were divided into four groups based on their serum betaine levels using the quartile method, and the levels of m^6^A modification in HCC tissues of these four groups (Q1 for quartile 1 group, Q2 for quartile 2 group, Q3 for quartile 3 group, Q4 for quartile 4 group) were detected. (B-E) The effects of betaine exposure on the m^6^A modification in HCC cells and PDX tumor tissues were detected using dot blot and quantified using the EpiQuik m^6^A methylation quantification kit, respectively. (F-G) After being exposed to betaine, the concentration of SAM in HCC cells and PDX tumor tissues was detected by a SAM ELISA kit. (H) HCC cells were treated with various concentrations of SAM (0, 25, 50, and 100 μM), and the concentration of SAM in HCC cells was detected by a SAM ELISA kit. (I-J) HCC cells were treated with various concentrations of SAM, and the m^6^A modification in HCC cells was detected using dot blot and quantified using the EpiQuik m6A methylation quantification kit, respectively. (K-N) HCC cells were pre-transfected with siBHMT, and then subjected to betaine or combined betaine and SAM treatments. The SAM levels, m^6^A modification, and LC3 I/II protein levels in HCC cells were detected via SAM ELISA kit, dot blot, EpiQuik m^6^A methylation quantification kit, and WB assay, respectively. *P < 0.05, **P < 0.01, ***P < 0.001.

As one of the pivotal epigenetic modifications in eukaryotes, m^6^A methylation has been reported to play a role in the activation of autophagy^29^, ^38^. Interestingly, betaine has been shown to act as an important methyl donor source for the generation of SAM. Consequently, we further explored whether betaine could promote autophagy in HCC cells via SAM-mediated m^6^A modification. In GLCC cohort, we found that HCC patients with a high level of serum betaine had an increased m^6^A content in HCC tissues (Figure 5A). Similarly, betaine treatment significantly elevated m^6^A content in HCC cells and PDX tumors (Figure 5B-E), while inhibition of autophagy, either through treatment with HCQ or transfection with shATG5, has no effect on m^6^A modification (Figure S8A-F).

Subsequently, we investigated the role of SAM in betaine-induced m^6^A modification in HCC. We found that betaine treatment markedly increased SAM levels in HCC cells and PDX tissues (Figure 5F-G), but no changes were observed when suppressing autophagy (Figure S8G-I). Moreover, SAM promoted m^6^A modification in a dose-dependent manner in HCC cells (Figure 5H-J). However, silencing of BHMT not only decreased the production of SAM but also inhibited the m^6^A modification in HCC cells, while additional SAM supplementation counteracted the downregulation effect of BHMT (Figure 5K-M, Figure S4C and S4I). Furthermore, our results demonstrated that knockdown of BHMT inhibited the expression of LC3 II, reduced the number of autophagosomes, and decreased LC3 puncta, whereas SAM treatment significantly reversed these effects (Figure 5N and Figure S9A -D). Therefore, our results imply that betaine activates autophagy in HCC via SAM-mediated m^6^A modification.

### Betaine activates autophagy by promoting the expression of ATG3 in a SAM/m^6^A-dependent manner

**Figure 6:**
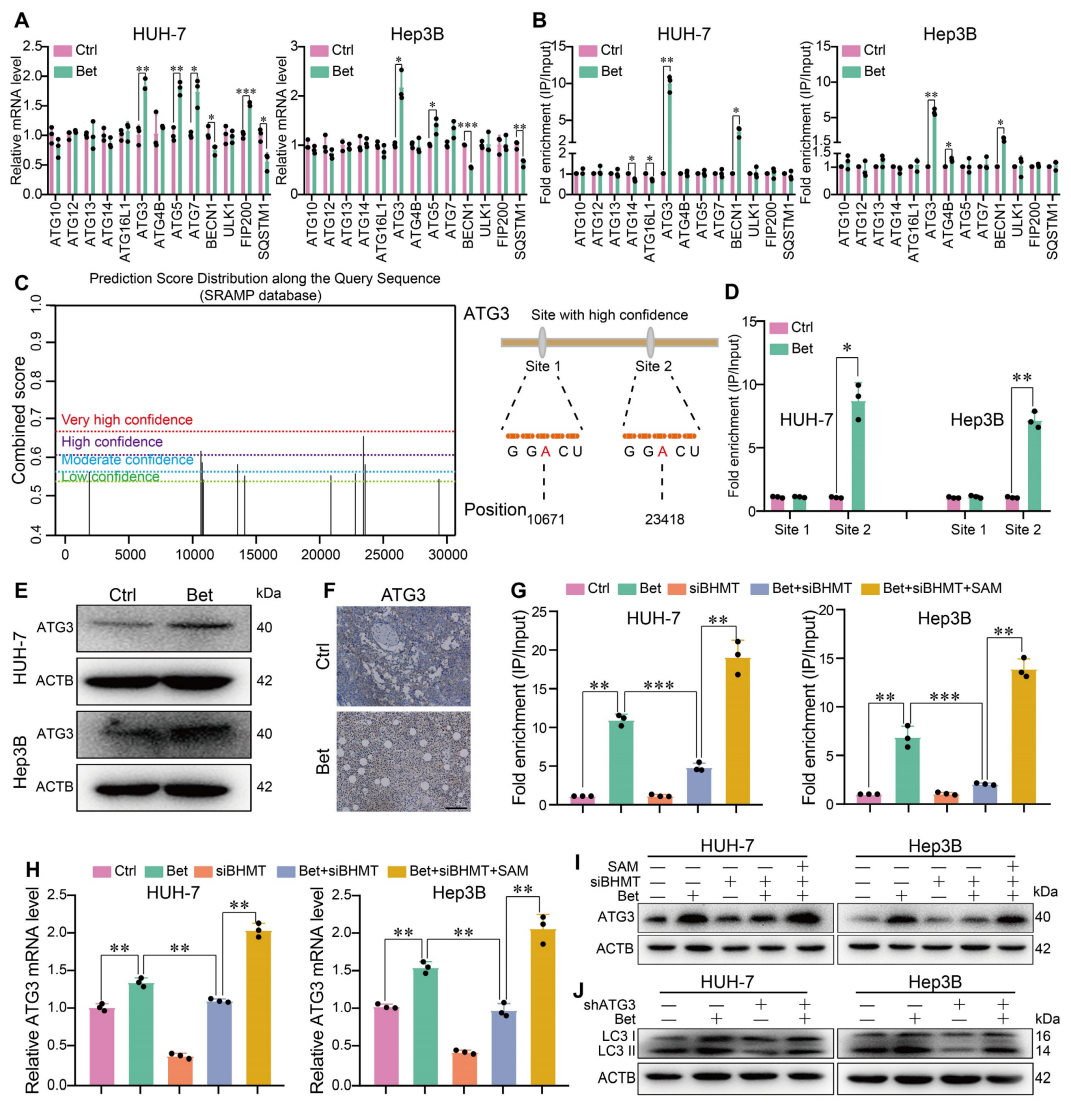
** Betaine activates autophagy via SAM-mediated m6A modification on ATG3 mRNA.** (A) The effects of betaine exposure on the expression levels of autophagy-related genes in HCC cells were detected by qPCR analysis. (B) After treatment with betaine, MeRIP-qPCR assay was performed to measure the level of m^6^A-modified ATGs in HCC cells. (C) m^6^A modification sites in ATG3 mRNA were predicted via SRAMP database and those with high confidence were selected for further investigation. (D) After treatment with betaine, the level of m^6^A modification in these two sites of ATG3 mRNA in HCC cells was detected using MeRIP-qPCR assay. (E-F) After treatment with betaine, the expression levels of ATG3 protein in HCC cells and PDX tumor tissues were measured using WB and IHC assays (Scale bar = 100 μm), respectively. (G-I) HCC cells were pre-transfected with siBHMT, and then were subjected to betaine or combined betaine and SAM treatments. The level of m^6^A modification in ATG3 mRNA, as well as the expression levels of ATG3 mRNA and protein, were detected using MeRIP-qPCR, qPCR, and WB assays, respectively. (J) HCC cells were pre-transfected with shATG3, and then were subjected to betaine treatment, the expression levels of LC3 I/II proteins were detected using WB assay. *P < 0.05, **P < 0.01, ***P < 0.001.

The process of autophagy activation is tightly regulated by a class of autophagy- related genes (ATGs). To clarify the target genes of betaine, we measured the m^6^A modification of ATGs in betaine-treated HCC cells. Initially, qPCR results indicated that among 13 key ATGs, only ATG3 and ATG5 mRNA were consistently upregulated, while SQSTM1 and BECN 1 were downregulated in HUH-7 and Hep3B cell lines after betaine exposure (Figure 6A). MeRIP-qPCR results demonstrated that betaine treatment increased the m^6^A modification on ATG3 and BECN 1 mRNA in two cell lines (Figure 6B), suggesting that betaine-mediated m^6^A modification may activate autophagy via ATG3. Meanwhile, MeRIP-qPCR further confirmed that the m^6^A modification at the predicted site 2 of ATG3 mRNA was significantly elevated in Bet group, while no significant change was observed in site 1(Figure 6C-D). In addition, WB and IHC assays illustrated that betaine exposure markedly enhanced the expression of ATG3 protein (Figure 6E-F).

Then, we explored whether the increase in ATG3 expression induced by betaine was attributed to SAM-mediated m^6^A modification. Results found that BHMT silencing not only inhibited m^6^A modification on ATG3 mRNA but also attenuated the expression of ATG3 mRNA and protein in HCC cells, while SAM supplementation reversed those effects (Figure 6G-I). Furthermore, silencing of ATG3 impaired the activation of autophagy by betaine in HCC cells (Figure 6J, Figure S4D-E, S4J-K, and S10A-D). Thus, our results demonstrate that betaine activates autophagy by upregulating ATG3 via SAM-mediated m^6^A modification.

### Betaine enhances the stability of ATG3 mRNA in a SAM/m^6^A/YTHDF1-dependent manner

**Figure 7:**
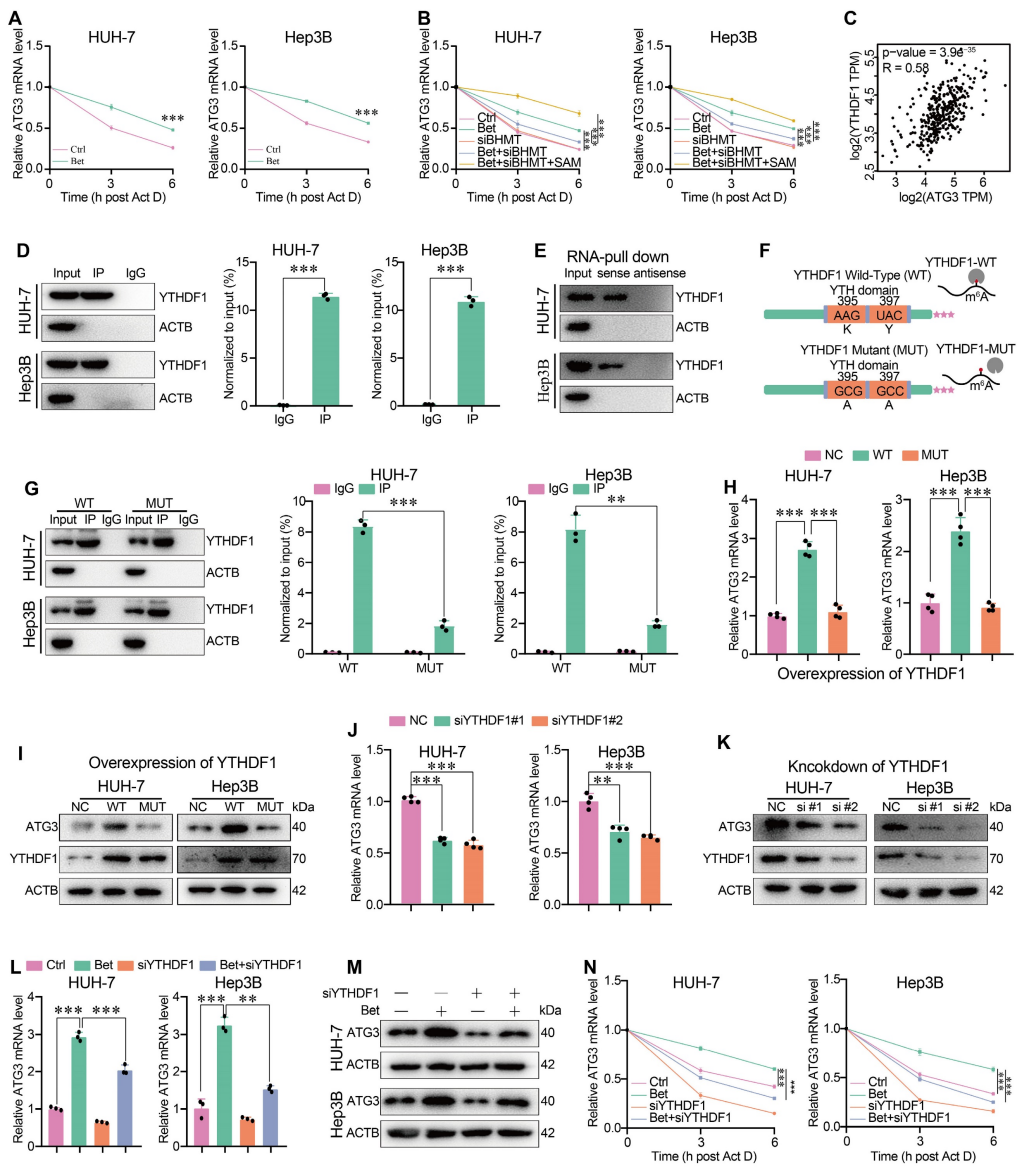
** Betaine promotes ATG3 mRNA stability via SAM/m6A/YTHDF1-dependent manner.** (A) After treatment with betaine, HCC cells were treated with 5 μg/mL of Act D for 0, 3, and 6 h, respectively. The expression levels of ATG3 mRNA at these different time points were detected using qPCR assay. (B) After pre-transfection with siBHMT, HCC cells were subjected to betaine or combined betaine and SAM treatments. Then, 5 μg/mL of Act D was added for further treatment for 0, 3, and 6 h, respectively. The expression levels of ATG3 mRNA at these different time points were detected using qPCR assay. (C) The correlation between ATG3 and YTHDF1 in HCC tissues was assessed using online GEPIA 2 database. (D) The interaction between ATG3 mRNA and YTHDF1 protein was analyzed using RIP-qPCR assay, and the RIP-derived protein and ATG3 mRNA in HCC cells were detected by WB and qPCR assays, respectively. (E) The interaction between ATG3 mRNA and YTHDF1 protein was confirmed by RNA pull-down assay, and the protein derived from the RNA pull-down assay in HCC cells was detected using WB assay. (F) Schematic graph of the YTHDF1 wild-type (YTHDF1-WT) and the mutant of YTHDF1 m^6^A-binding pocket (YTHDF1-MUT) construction. (G) HCC cells were pre-transfected with YTHDF1-WT and YTHDF1-MUT overexpressed plasmids, and the interaction between ATG3 mRNA and YTHDF1 was analyzed via RIP-qPCR assay. (H and I) HCC cells were transfected with YTHDF1-WT and YTHDF1-MUT overexpressed plasmids, and the expression levels of ATG3 mRNA as well as the levels ATG3 and YTHDF1 proteins were detected using qPCR and WB assays, respectively. (J and K) HCC cells were transfected with siYTHDF1, and the expression levels of ATG3 mRNA as well as the levels of ATG3 and YTHDF1 proteins were detected using qPCR and WB assays, respectively. (L-M) HCC cells were pre-transfected with siYTHDF1, and then were subjected to betaine treatment. The expression levels of ATG3 mRNA and protein were detected using qPCR and WB assays, respectively. (N) After pre-transfection with siYTHDF1, HCC cells were subjected to betaine treatment. Then, 5 μg/mL of Act D was added for further treatment for 0, 3, and 6 h, respectively. The expression levels of ATG3 mRNA at these different time points were detected using qPCR assay. **P < 0.01, ***P < 0.001.

It has been reported that the m^6^A modification can influence the stability, translation, splicing, decay, and transport of RNAs through specific reader proteins 39. To elucidate which reader mediates the upregulation of ATG3 under betaine treatment via m^6^A-dependent manner, we initially focused on the stability of ATG3 mRNA, given the observed elevation of ATG3 mRNA levels under betaine treatment. qPCR assays revealed that betaine significantly restored the level of ATG3 mRNA after Act D treatment compared to the control (Ctrl) group (Figure 7A), and silencing of BHMT attenuated the upregulation of ATG3 mRNA level under betaine treatment (Figure 7B). However, additional SAM supplementation significantly reversed the effect of BHMT knockdown on betaine-increased ATG3 mRNA stability (Figure 7B), suggesting that betaine could enhance the stability of ATG3 mRNA via SAM-mediated m^6^A modification. Next, bioinformatic analysis found that, among the m^6^A-related readers that mediates the stability of RNAs, YTHDF1 was significantly positively correlated with ATG3 expression in HCC tissues (Figure 7C). Therefore, we further investigated the role of YTHDF1 in mediating ATG3 mRNA stability. RIP-qPCR and RNA pull-down results demonstrated that YTHDF1 could directly interact with ATG3 mRNA (Figure 7D-E).

To confirm that the interaction between YTHDF1 protein and ATG3 mRNA was m^6^A-dependent, we genetically impaired the m^6^A-binding pockets of YTHDF1 by mutating the K395 and Y397 residues within the YTH domain (Figure 7F). RIP-qPCR results indicated that ATG3 mRNA was significantly immunoprecipitated from HCC cells transfected with YTHDF1-WT, while the interaction between YTHDF1-MUT and ATG3 mRNA was significantly decreased, implying the pivotal role of m^6^A-binding pocket of YTHDF1 in its interaction with ATG3 mRNA (Figure 7G). Moreover, we found that YTHDF1-WT, but not YTHDF1-MUT, significantly upregulated the levels of ATG3 mRNA and protein (Figure 7H-I). Finally, we found that knockdown of YTHDF1 not only decreased ATG3 mRNA and protein levels, but also impaired the enhancing effect of betaine on ATG3 mRNA and protein levels as well as ATG3 mRNA stability (Figure 7J-N and Figure S4F). Thus, our data demonstrate that betaine could promote ATG3 mRNA stability via a SAM/m^6^A/YTHDF1-dependent mechanism.

### Silencing of ATG3 impairs the effects of betaine on the stem cell-like properties of HCC cells

**Figure 8:**
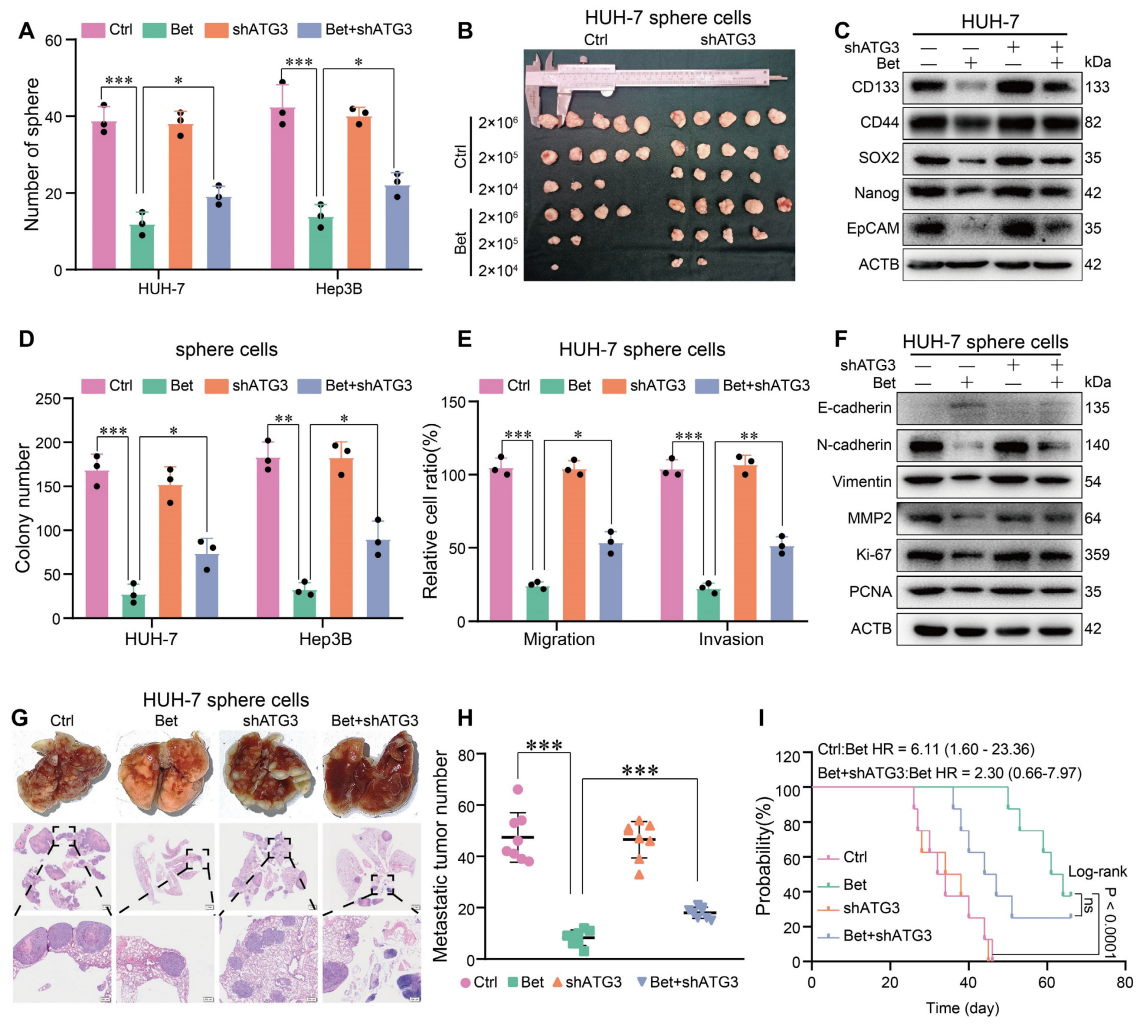
** betaine inhibits the stem cell-like properties of HCC via ATG3.** (A) HUH-7 cells were pre-transfected with shATG3 and subjected to betaine treatment. The tumor sphere formation ability of HUH-7 cells under different treatments was measured. A statistical graph is shown. (B) HUH-7 cells were pre-transfected with shATG3 and subjected to betaine treatment for generation of sphere cells. Then, HUH-7 sphere cells were diluted in a gradient and injected subcutaneously into nude mice. The number of tumors in each group was counted after 30 days. The injected cell quantity and tumor formation frequency (n = 5 per group) were shown. (C) HUH-7 cells were pre-transfected with shATG3, and then subjected to betaine treatment. The expression levels of CD133, CD44, SOX2, Nanog, and EpCAM proteins in HUH-7 cells were detected using WB assay. (D-E) HUH-7 cells were pre-transfected with shATG3 and subjected to betaine treatment for generation of sphere cells. After being dissociated and diluted, sphere cells were subjected to colony formation, and transwell migration and invasion assays. Statistical graphs are shown. (F) WB analysis of the expression levels of E-cadherin, N-cadherin, Vimentin, MMP2, Ki-67, and PCNA proteins in HUH-7 sphere cells under different treatments. (G) HUH-7 cells were pre-transfected with shATG3 and subjected to betaine treatment for generation of sphere cells. After being dissociated and diluted, 5×10^6^ of cells were injected into the tail vein of nude mice. After 66 days of injection, mice were humanely euthanized. Images of lung metastatic nodules and H&E staining were shown (scale bar = 1 mm for upper panel, and 200 μm for lower panel). (H) Quantitative results of lung metastatic nodules in different groups. (I) Kaplan-Meier survival curve analysis of nude mice injected with HCC cells in different groups (n = 8/group). HR: Hazard Ratio; ns: not significant. *P < 0.05, **P < 0.01, ***P < 0.001.

Given the crucial role of ATG3 in betaine-activated autophagy, we conducted rescue assays to investigate the effect of ATG3 on the suppression of stem cell-like properties in HCC cells induced by betaine. Results from sphere formation, *in vitro*, and *in vivo* limiting dilution assays revealed that silencing of ATG3 significantly impaired the inhibitory effects of betaine on the stem cell-like properties of HCC cells (Figure 8A-B and Figure S11A-D). Additionally, the knockdown of ATG3 markedly attenuated the downregulation of stemness-related markers induced by betaine treatment (Figure 8C and Figure S11E).

Furthermore, colony formation, transwell, WB, and* in vivo* metastasis assays demonstrated that ATG3 silencing mitigated the effects of betaine on malignant cellular phenotypes, as evidenced by increased colony formation, enhanced migration and invasion, upregulated protein markers, and elevated metastatic nodules (Figure 8D-H and Figure S11F-I). Although there was no significant difference in the survival duration between betaine group and betaine+shATG3 group, we can still observe a decreased trend of survival time in betaine+shATG3 group (Figure 8I). In summary, our findings illustrate that betaine inhibits the stem cell-like properties of HCC cells through the upregulation of ATG3 in SAM/m^6^A/YTHDF1-dependent manner.

## Discussion

Although a few studies have reported the protective effects of betaine on liver diseases, its roles in HCC progression, particularly in the context of stem cell-like properties, remain unknown. Furthermore, the mechanism underlying how betaine exhibits its anti-tumor effects has been poorly investigated. In this study, we found that betaine significantly inhibited the stem cell-like properties of HCC cells, as well as suppressed the growth and metastasis of HCC sphere cells both *in vitro* and *in vivo*. Moreover, the anti-tumor effects of betaine were markedly attenuated by inhibiting autophagy. Mechanistically, betaine promoted m^6^A modification on ATG3 mRNA by generating SAM, while YTHDF1 recognized and bound to ATG3 mRNA to enhance its stability. Collectively, betaine inhibited the stem cell-like properties of HCC by activating autophagy via SAM/m^6^A/YTHDF1-mediated enhancement on ATG3 stability.

Despite great advancements in diagnosis and treatment, HCC remains the third leading cause of cancer-associated death worldwide. Recent studies have consistently shown that the primary contributors to poor prognosis in HCC patients are recurrence and distant metastasis, resulting in a significant reduction in overall and cancer-specific survival times 40-42. Hence, targeting the risk factors associated with the recurrence and metastasis provides an opportunity for improving the prognosis of HCC. Cancer stem cells (CSCs) are a small population of cancer cells with high capacities of self-renewal, differentiation, and carcinogenic potential. They have been implicated in cancer recurrence and metastasis 43, 44. Patients with elevated levels of CSCs-related genes tend to experience a worse prognosis, whereas suppression of stem cell-like properties can inhibit recurrence and metastasis. Recently, a few phytochemicals have been reported to possess the ability to hinder the stem cell-like properties of cancer cells. For example, curcumin has demonstrated its ability to inhibit breast cancer cell metastasis by suppressing stem cell-like properties 45. Similarly, pharmacological ascorbate has been shown to reduce these properties, thereby preventing recurrence and systemic metastasis after surgery 46. Betaine, a natural compound in many foods, has shown protective roles against liver cancer. For example, our previous studies found that high dietary betaine intake is associated with a reduced risk of primary liver cancer, and betaine treatment alleviated DEN-induced liver cancer in rats 20, 23. Meanwhile, Oliva *et al.* reported that betaine administration inhibited the growth of murine HCC cells 47. However, the impact of betaine on the late-stage-related phenotype of HCC, which significantly influences the prognosis of HCC patients, and the mechanisms behind its anti-tumor effects remain uninvestigated. In this study, we found that HCC patients with higher serum betaine levels exhibited lower expression of stemness-related genes. *In vitro* and *in vivo* experiments further demonstrated that betaine treatment significantly attenuated the stem cell-like properties of HCC cells. Moreover, mice treated with betaine exhibited reduced lung metastatic nodules and prolonged survival times.

Autophagy is a highly conserved catabolic process in eukaryotes that is crucial for maintaining cellular homeostasis 48. The process, known as autophagic flux, involves a series of ATGs orchestrating the formation of double-membrane autophagosomes to sequester cellular components. These autophagosomes then fuse with lysosomes to degrade and recycle cargo, thereby supporting cancer cell survival 10. Numerous studies have shown that the activation of autophagy contributes to the acquisition of stem cell-like properties in cancer cells through regulation of stemness-related genes or modulation of tumor microenvironments 49, 50. Consequently, autophagy plays a significant role in the stem cell-like properties of cancer cells. However, recent research has unveiled a contrasting role for autophagy activated by phytochemicals in stem cell-like properties. For example, curcumin inhibits colorectal cancer stem cells by activating autophagy via suppressing TFAP2A/ECM pathway 51. Similarly, posaconazole attenuates the stem cell-like properties by inducing autophagy in glioblastoma 44. Although betaine has been reported to exert protective effects against liver diseases by activating autophagy 24, 25, its impact on the stem cell-like properties of HCC cells remains unknown. In the present study, we demonstrate that betaine activates autophagic flux to inhibit the stem cell-like properties of HCC cells both *in vitro* and *in vivo*, while inhibition of autophagy not only accelerates the growth and metastasis of HCC cells but also shortens the survival time of mice. Overall, our findings indicate that betaine inhibits the stem cell-like properties of HCC cells by activating autophagic flux.

As an important phytochemical, the protective roles of betaine have been well demonstrated in several liver diseases, but the underlying mechanisms remain poorly understood. Betaine serves two primary biological functions. Firstly, as an effective osmolyte, it regulates intercellular osmotic pressure akin to other electrolytes 34. Secondly, betaine acts as a crucial methyl donor, producing SAM via BHMT, thereby regulating genomic methylation 34. For example, several studies have linked betaine's protective effects to its modulation of DNA methylation in rat models 52-55. In addition, recent attention has focused on betaine's regulation of RNA methylation, particularly m^6^A modification 56-58. m^6^A modification is a prevalent epi-transcriptional alteration in mRNA that governs various mRNA processes, including splicing, nuclear transport, decay, stability, and translation, via specific readers. Increasing evidence highlights the crucial role of m^6^A modulation in autophagy activation, particularly its direct regulation of ATGs 28. For example, YTHDF1 activates autophagy by stabilizing BECN1, leading to hepatic stellate cell ferroptosis and ameliorating liver fibrosis in murine models 59. METTL14 suppresses the development of oral squamous cell carcinoma by activating autophagy via m^6^A/IGF2BP2-dependent modification on FIP200 stability 60. Our study demonstrates that betaine treatment enhances total m^6^A modification in HCC cells in a BHMT/SAM-dependent manner, while inhibition of SAM by BHMT silencing significantly attenuates betaine-induced autophagic flux. Further investigation unveiled that betaine activates autophagic flux by promoting m^6^A modification on ATG3 mRNA, thereby upregulating its mRNA and protein expression. Conversely, ATG3 silencing impairs betaine's inhibitory effects on the stem cell-like properties of HCC cells. These data indicate that betaine inhibits the stem cell-like properties of HCC cells by activating ATG3-dependent autophagy via m^6^A modification.

After m^6^A modification, specific readers govern the fate of mRNAs. Given the increased m^6^A modification on ATG3 mRNA and the upregulation of its mRNA level, we investigated the mRNA stability under betaine treatment. RNA stability assay found that betaine significantly enhanced the stability of ATG3 mRNA. Silence of BHMT attenuated the effect of betaine on ATG3 mRNA stability, while additional SAM treatment reversed this effect, indicating that betaine promoted ATG3 expression by enhancing its stability via m^6^A modification. To date, several m^6^A-related readers, such as YTHDF1 and IGF2BP1/2/3, are known to enhance the stability of mRNAs. In the present study, we found that YTHDF1 was positively correlated with ATG3 expression in HCC. RIP-qPCR and RNA pull-down confirmed the interaction between YTHDF1 protein and ATG3 mRNA. Previous study has demonstrated that YTHDF1 recognizes and binds to the m^6^A site of targeted mRNA via m^6^A-binding pockets 28. To further investigate whether the interaction between YTHDF1 and ATG3 was m^6^A dependent, we genetically mutated YTHDF1 at residues K395 and Y397. In line with previous findings, we found that YTHDF1 enhanced the stability of ATG3 mRNA in an m^6^A-dependent manner.

This study utilized different HCC line lines and animal models to investigate the effects of betaine on the stem cell-like properties of HCC. The consistency of the results provided a reliable, generalized, and comprehensive understanding of our study. However, there is a limitation in this study. While our study examines the effects of betaine treatment on the growth of PDX tumors and the changes of specific histological features such as nuclear atypia, mitosis, and the nuclear-to-cytoplasmic ratio, we did not observe significant differences in tumor grading, categorizing tumors into well, moderately, and poorly differentiated grades.

## Conclusion

In this study, we found that betaine increased the m^6^A modification on ATG3 mRNA by producing SAM via BHMT. Subsequently, YTHDF1 was found to recognize and bind to ATG3 mRNA, enhancing its stability in an m^6^A-dependent manner. Consequently, the upregulated ATG3 activated autophagy, thereby inhibiting the stem cell-like properties of HCC cells. Our findings provide a novel understanding of the anti-tumor effects of betaine on HCC and its underlying mechanism, potentially expanding therapeutic options for HCC patients.

## Supplementary Material

Supplementary methods, figures and tables.

## Abbreviations

ATGs: autophagy related genes
BHMT: betaine-homocysteine methyltransferase
CCK-8: cell counting kit-8
ELISA: enzyme-linked immunosorbent
EMT: epithelial-mesenchymal transition
GLCC: Guangdong liver cancer cohort
HCC: hepatocellular carcinoma
HE: hematoxylin & eosin
IF: immunofluorescence
IHC: immunohistochemistry
MeRIP-qPCR: Methylated RNA Immunoprecipitation-qPCR
m^6^A: N^6^-methyladenosine
PDX: patient derived xenografts
RIP-qPCR: RNA immunoprecipitation-qPCR
SAM: S-adenosylmethionine
TEM: transmission electronic microscopy
