# Supplementary material for: Betaine inhibits the stem cell-like properties of hepatocellular carcinoma by activating autophagy via SAM/m6A/YTHDF1-mediated enhancement on ATG3 stability
Source: Theranostics. 2025 Jan 6;15(5):1949–65. doi: 10.7150/thno.102682 (PMC11780527; doi:10.7150/thno.102682)
Supplement: Supplementary file 1 — Supplementary methods, figures and tables. [file thnov15p1949s1.pdf]

Supplementary methods

Cell culture

The HCC cell lines (HUH-7 and Hep3B) were obtained from the National Collection of Authenticated Cell Cultures (Shanghai, China). Briefly, HUH-7 and Hep3B cells were cultured in DMEM and MEM containing 10% FBS and 1% antibiotic-antimycotic, respectively. Cells were incubated in a 37 ℃ incubator with 5% CO_2. Cells were authenticated before the experiment.

Transient transfection

HCC cells were planted into a 6-well plate at a density of 75% confluence. Then, ATG5-, ATG3-, YTHDF1-, and BHMT-specific small interfering RNA (siRNA) and negative control (NC) transfection were mixed with Opti-MEM and lipofectamine 3000, respectively. After being incubated for 20 min, the mixture was subjected to HCC cells and cultured for 8 h. Subsequently, the mixture was removed and fresh medium was added for further cultivation for 48 h. Finally, cells were harvested for subsequent experiments. The sequence of the siRNAs and NC was listed in Table S3.

Construction of stable transfected cell line

To obtain a stable transfected cell line, the recombined lentiviruses harboring ATG5 and ATG3 short hairpin RNA (shRNA) as well as scrambled lentiviral constructs (shNC) were designed and constructed by GenePharm company (Soochow, China). Briefly, after HCC cells in 12-well plate reached a confluence of 35%, lentivirus was added into each well and cultured for 8 h. Then, 1 mL of fresh medium was added and further

incubated for 48 h. Afterwards, the medium was removed and fresh medium containing 2.5 μg/mL of puromycin was added for 14 days to select stable cell lines. The sequences of shRNA (ATG5 and ATG3) and shNC (NC) were listed in Table S4.

CCK-8 assay

CCK-8 assay was conducted to detect the effect of betaine on the viability of HCC cells. Briefly, 5000 HCC cells were planted into a 96-well plate and subjected to different concentrations of betaine for 24 h. Then, the medium was removed and 90 μL of fresh medium along with 10 μL of CCK-8 reagent were added into each well. After being incubated at 37 °C for 2 h, the absorbance of each well was detected by a microplate reader at 450 nm. The IC50 values of betaine against HCC cells were calculated according to the concentration and absorbance.

Colony formation assay

Colony formation assay was performed to detect the cell clonogenic ability. Briefly, HCC spheres with different treatments were dissociated into a single cell using accutase, and 400 cells suspended in 2 mL of medium were planted into a 12-well plate and incubated for 10 days. Then, the medium was removed, and the cells were washed with PBS and fixed with 4% paraformaldehyde. Subsequently, the cells were stained with crystal violet for 30 min and washed. After drying, images were captured and the colonies were counted.

Transwell migration and invasion assays

Transwell migration and invasion assays were conducted to measure cell motility. First, HCC spheres with different treatments were dissociated into a single cell by accutase. In the transwell migration assay, 5000 cells suspended in 100 μL of fresh medium were added into the upper chamber, while 500 μL of fresh medium was added into the lower chamber. After being cultured for 48 h, cells in the upper chamber were gently removed. Then, cells in the lower chamber were fixed with 4% paraformaldehyde for15 min and stained with crystal violet for 20 min. Finally, images were captured using a light microscope, and the migrated cells were calculated by ImageJ software.

For the transwell invasion assay, 50 μL of Matrigel (Corning, New York, USA) was pre-coated in the upper chamber for 2 h in the incubator. Then, 10,000 cells diluted in 100 μL of fresh medium were planted into the upper chamber. The remaining procedures followed the migration assay.

Western blots

Total proteins in HCC cells and tissues were extracted using RIPA lysis buffer containing 1% PMSF and 1% protease inhibitor. After quantification and denaturation, about 15-30 μg of proteins were added into sodium dodecyl sulphate-polyacrylamide gel electrophoresis (SDS-PAGE) for separation. Then, the proteins were transferred to a polyvinylidene fluoride (PVDF) membrane for 2 h. Afterwards, the membrane was blocked with 5% skim milk at room temperature for 1 h and washed with TBST for 15 min. After incubation with primary antibodies overnight at 4 °C, the membrane was

washed and incubated with secondary antibody at room temperature for 2 h. Subsequently, the membrane was washed and detected by enhanced chemiluminescence. The band density of proteins was measured by ImageJ software. Details of antibodies were listed in Table S5.

RNA isolation, cDNA synthesis, and quantitative polymerase chain reaction

Total RNA in HCC cells and tissues was isolated using a commercial TransZOL kit. After quantification, about 100 ng of RNA was subjected to cDNA synthesis using TransScript® II One-Step gDNA Removal and cDNA Synthesis SuperMix kit (TransGen Biotech, Beijing, China) according to the guideline of manufacturer. Then, relative gene expression was determined via quantitative polymerase chain reaction (qPCR) method using a TransStart Top Green qPCR SuperMix Kit (TransGen Biotech) following the manufacturers' instructions. The expression of genes was normalized to 18S rRNA and calculated using the $2^{-\Delta\Delta CT}$ method. The primer sequences were listed in Table S6.

Enzyme-linked immunosorbent assay

The level of SAM in PDX tumor tissues and HCC cells was detected using a commercial enzyme-linked immunosorbent (ELISA) kit (JonIn, Shanghai, China) according to the manufacturer's protocol.

Bioinformatic analysis

The correlation of ATG3 and YTHDF1 in HCC tissues was analyzed using the online GEPIA 2 software according to its instruction.

Hematoxylin & Eosin staining

After the tissues were fixed with 4% paraformaldehyde, embedded with paraffin, and sectioned, about 4 μm thick of tissues were subjected to Hematoxylin & Eosin (H&E) staining. The images of H&E-stained tissues were captured using a light microscope. For tail vein-lung metastasis assay, the metastatic nodules were counted under the microscope.

Immunohistochemistry assay

For the immunohistochemistry (IHC) assay, 4 μm thick of tissues were subjected to xylene for deparaffinization and graded ethanol for rehydration. Then, tissues were boiled in EDTA (pH = 8.0) for 30 min for antigen retrieval. Afterward, 3% H_2O_2 was used to quench the endogenous peroxidase. Subsequently, the tissues were blocked with 1% BSA for 20 min at room temperature. After being washed with PBS for 3 times, the tissues were incubated with primary antibody overnight at 4 °C in a humid chamber. Then, tissues were washed with PBS and incubated with second antibodies at room temperature for 20 min in a humid chamber. After being washed, the tissues were stained with DAB reagent and hematoxylin. Finally, the tissues were sealed with neutral balsam and images were captured with a light microscope.

Immunofluorescence assay

For immunofluorescence (IF) assay, the procedures from deparaffinization to primary antibodies incubation followed the IHC assay. After being washed with PBS, the tissues were incubated with different species-derived secondary antibodies at 4 ℃ for 2 h at room temperature. Then, the tissues were incubated with DAPI (Beyotime) for 5 min and sealed with anti-fluorescence quenching buffer. Finally, images were captured using a fluorescence microscope.

Table S1. YTHDF1 wild type and mutant sequence

Gene	sequence
h-YTHDF1-wt	ATGTCGGCCACCAGCGTGGACACCCAGAGAACAAAAGGACAAGATAATAAAG<br>TACAAAATGGTTCGTTACATCAGAAGGATACAGTTCATGACAATGACTTTGAG<br>CCCTACCTTACTGGACAGTCAAATCAGAGTAACAGTTACCCCTCAATGAGCGA<br>CCCCTACCTGTCCAGCTATTACCCGCCGTCCATTGGATTTCCTTACTCCCTCAAT<br>GAGGCTCCGTGGTCTACTGCAGGGGACCCTCCGATTCCATACCTCACCACCTA<br>CGGACAGCTCAGTAACGGAGACCATCATTTTATGCACGATGCTGTTTTTGGGC<br>AGCCTGGGGGCCTGGGGAACAACATCTATCAGCACAGGTTCAATTTTTTCCCT<br>GAAAACCCTGCGTTCTCAGCATGGGGGACAAGTGGGTCTCAAGGTCAGCAGA<br>CCCAGAGCTCCGCGTATGGGAGCAGCTACACCTACCCCCCGAGCTCCCTGGGT<br>GGCACGGTGGTTGATGGGCAGCCAGGCTTTCACAGCGACACCCTCAGCAAGG<br>CCCCCGGGATGAACAGCCTGGAGCAGGGCATGGTTGGCCTGAAGATTGGGGA<br>CGTCAGCTCCTCCGCCGTCAAGACGGTGGGCTCTGTCGTCAGCAGCGTGGCA<br>CTGACTGGTGTCCTTTCTGGCAACGGTGGGACAAATGTGAACATGCCAGTTTC<br>AAAGCCGACCTCGTGGGCTGCCATTGCCAGCAAGCCTGCAAAACCACAGCCT<br>AAAATGAAAACAAAGAGCGGGCCTGTCATGGGGGGTGGGCTGCCCCCTCCAC<br>CCATAAAGCATAACATGGACATTGGCACCTGGGATAACAAGGGGCCTGTGCCG<br>AAGGCCCCAGTCCCCCAGCAGGCACCCTCTCCACAGGCTGCCCCACAGCCCC<br>AGCAGGTGGCTCAGCCTCTCCCAGCACAGCCCCCAGCTTTGGCTCAACCGCA<br>GTATCAGAGCCCTCAGCAGCCACCCCAGACCCGCTGGGTTGCCCCACGCAAC<br>AGAAACGCGGCGTTTGGGCAGAGCGGAGGGGCTGGCAGCGATAGCAACTCTC<br>CTGGAAACGTCCAGCCTAATTCTGCCCCCAGCGTCGAATCCCACCCCGTCCTT<br>GAAAAACTGAAGGCTGCTCACAGCTACAACCCGAAAGAGTTTGAGTGGAATC<br>TGAAAAGCGGGCGTGTGTTCATCATCAAGAGCTACTCTGAGGACGACATCCAC<br>CGCTCCATTAAGTACTCCATCTGGTGTAGCACAGAGCACGGCAACAAGCGCCT<br>GGACAGCGCCTTCCGCTGCATGAGCAGCAAGGGGCCCGTCTACCTGCTCTTCA<br>GCGTCAATGGGAGTGGGCATTTTTGTGGGGTGGCCGAGATGAAGTCCCCCGTG<br>GACTACGGCACCAGTGCCGGGGTCTGGTCTCAGGACAAGTGGAAGGGGAAGT<br>TTGATGTCCAGTGGATTTTTGTTAAGGATGTACCCAATAACCAGCTCCGGCACA<br>TCAGGCTGGAGAATAACGACAACAAACCGGTCACAAACTCCCGGGACACCCA<br>GGAGGTGCCCTTAGAAAAAGCCAAGCAAGTGCTGAAAATTATCAGTTCCTACA<br>AGCACACAACCTCCATCTTCGACGACTTTGCTCACTACGAGAAGCGCCAGGA<br>GGAGGAGGAGGTGGTGCGCAAGGAACGGCAGAGTCGAAACAAACAATGA
h-YTH	ATGTCGGCCACCAGCGTGGACACCCAGAGAACAAAAGGACAAGATAATAAAG<br>TACAAAATGGTTCGTTACATCAGAAGGATACAGTTCATGACAATGACTTTGAG<br>CCCTACCTTACTGGACAGTCAAATCAGAGTAACAGTTACCCCTCAATGAGCGA<br>CCCCTACCTGTCCAGCTATTACCCGCCGTCCATTGGATTTCCTTACTCCCTCAAT<br>GAGGCTCCGTGGTCTACTGCAGGGGACCCTCCGATTCCATACCTCACCACCTA<br>CGGACAGCTCAGTAACGGAGACCATCATTTTATGCACGATGCTGTTTTTGGGC<br>AGCCTGGGGGCCTGGGGAACAACATCTATCAGCACAGGTTCAATTTTTTCCCT

**DF1-K395A/Y397A**	GAAAACCCTGCGTTCTCAGCATGGGGGACAAGTGGGTCTCAAGGTCAGCAGA<br>CCCAGAGCTCCGCGTATGGGAGCAGCTACACCTACCCCCCGAGCTCCCTGGGT<br>GGCACGGTGGTTGATGGGCAGCCAGGCTTTCACAGCGACACCCTCAGCAAGG<br>CCCCCGGGATGAACAGCCTGGAGCAGGGCATGGTTGGCCTGAAGATTGGGGA<br>CGTCAGCTCCTCCGCCGTCAAGACGGTGGGCTCTGTCGTCAGCAGCGTGGCA<br>CTGACTGGTGTCCTTTCTGGCAACGGTGGGACAAATGTGAACATGCCAGTTTC<br>AAAGCCGACCTCGTGGGCTGCCATTGCCAGCAAGCCTGCAAAACCACAGCCT<br>AAAATGAAAACAAAGAGCGGGCCTGTCATGGGGGGTGGGCTGCCCCCTCCAC<br>CCATAAAGCATAACATGGACATTGGCACCTGGGATAACAAGGGGCCTGTGCCG<br>AAGGCCCCAGTCCCCCAGCAGGCACCCTCTCCACAGGCTGCCCCACAGCCCC<br>AGCAGGTGGCTCAGCCTCTCCCAGCACAGCCCCCAGCTTTGGCTCAACCGCA<br>GTATCAGAGCCCTCAGCAGCCACCCCAGACCCGCTGGGTTGCCCCACGCAAC<br>AGAAACGCGGCGTTTGGGCAGAGCGGAGGGGCTGGCAGCGATAGCAACTCTC<br>CTGGAAACGTCCAGCCTAATTCTGCCCCCAGCGTCGAATCCCACCCCGTCCTT<br>GAAAAACTGAAGGCTGCTCACAGCTACAACCCGAAAGAGTTTGAGTGGAATC<br>TGAAAAGCGGGCGTGTGTTCATCATCgcgAGCgccTCTGAGGACGACATCCACC<br>GCTCCATTAAGTACTCCATCTGGTGTAGCACAGAGCACGGCAACAAGCGCCTG<br>GACAGCGCCTTCCGCTGCATGAGCAGCAAGGGGCCCGTCTACCTGCTCTTCAG<br>CGTCAATGGGAGTGGGCATTTTTGTGGGGTGGCCGAGATGAAGTCCCCCGTGG<br>ACTACGGCACCAGTGCCGGGGTCTGGTCTCAGGACAAGTGGAAGGGGAAGTT<br>TGATGTCCAGTGGATTTTTGTTAAGGATGTACCCAATAACCAGCTCCGGCACAT<br>CAGGCTGGAGAATAACGACAACAAACCGGTCACAAACTCCCGGGACACCCAG<br>GAGGTGCCCTTAGAAAAAGCCAAGCAAGTGCTGAAAATTATCAGTTCCTACAA<br>GCACACAACCTCCATCTTCGACGACTTTGCTCACTACGAGAAGCGCCAGGAG<br>GAGGAGGAGGTGGTGCGCAAGGAACGGCAGAGTCGAAACAAACAATGA

Table S2. Serum betaine concentrations of HCC patients

Number	Age	Gender	Betaine level (μmol/L)
NO0684	29	1	21
NO0239	53	1	33
NO0841	37	1	35
NO0658	44	1	39
NO0234	64	1	40
NO0944	65	1	41
NO0029	34	1	42
NO0952	66	0	43
NO0230	38	0	44
NO0969	60	1	46
NO0010	69	1	46
NO0665	58	1	47
NO0020	76	1	47
NO0023	50	1	49
NO0867	67	1	49
NO0009	56	1	49
NO0835	71	0	50
NO0288	36	1	50
NO0645	55	1	51
NO0748	63	1	53
NO0238	56	1	53
NO0006	46	1	54
NO0806	59	0	55
NO0879	47	1	55
NO0046	41	1	55
NO0882	54	1	56
NO0685	35	1	58
NO0005	55	1	59
NO0307	35	1	59
NO0818	45	1	60
NO0007	62	1	61
NO0917	61	1	61
NO0249	58	1	62
NO0951	61	1	63
NO0279	40	1	63
NO0842	42	1	64
NO0244	73	0	64
NO0938	50	1	65
NO0693	57	0	67
NO0843	49	1	68
NO0826	63	1	69
NO0866	41	1	69

NO0731	55	1	69
NO0729	49	1	70
NO0024	47	1	70
NO0981	58	1	74
NO0833	41	1	76
NO0038	65	0	76
NO0002	80	1	77
NO0014	71	1	79
NO0669	40	1	80
NO0751	52	1	80
NO0719	64	1	80
NO0034	50	1	81
NO0809	36	1	82
NO0688	20	0	82
NO0752	65	1	82
NO0812	35	1	83
NO0710	49	1	84
NO0229	47	1	84
NO0942	54	0	87
NO0780	53	1	91
NO0039	44	1	91
NO0016	46	1	92
NO0289	33	1	93
NO0650	51	1	100
NO0001	58	1	104
NO0690	37	1	108
NO0698	59	1	109
NO0877	64	1	131

Table S3. Small interference RNA sequences

siRNA	Sequence
**Scrambled**	5’-UUCUCCGAACGUGUCACGUTT-3’
**siATG5 #1**	5’- GGUUUGGACGAAUUCCAACUUGUUU -3’
**siATG5 #2**	5’- GAUCACAAGCAACUCUGGAUGGGAU-3’
**siATG3 #1**	5’-CUCAAGGAAUCAAAGUUUATT-3’
**siATG3 #2**	5’- GGGUAGAUACAUAUCACAATT -3’
**siBHMT #1**	5’- GCAGCUGAAAGAGCUCUUUTT-3’
**siBHMT #2**	5’- GCUGUGGAUUUGAGCCCUATT-3’
**siYTHDF1 #1**	5’- GGAAACGUCCAGCCUAAUUTT-3’
**siYTHDF1 #2**	5’- CCUACGGACAGCUCAGUAATT-3’

Table S4. shRNA sequences

shRNA	Sequence
**LV16NC**	5'-TTCTCCGAACGTGTCACGT-3'
**shATG5 #1**	5'- CAGCTCTTCCTTGGAACATCA -3'
**shATG5 #2**	5'- ATGAGACAAGAAGACATTAGT -3'
**shATG3 #1**	5'-CATATCACAACACAGGTATTA-3'
**shATG3 #2**	5'-GTACATCACTTACGACAAATA-3'

Table S5. Details of antibodies

Antibody	Company	Catalog	Concentration
**E-cadherin**	ABclonal	A20798	1:1000
**N-cadherin**	ABclonal	A19083	1:2000
**Vimentin**	ABclonal	A19607	1:10000
**MMP2**	ABclonal	A6247	1:500
**CD133**	Cell Signaling technology	64326	1:1000 for WB, 1:500 for IF
**CD44**	ABclonal	A19020	1:1000
**EpCAM**	ABclonal	A19301	1:10000
**SOX2**	Cell Signaling technology	23064	1:1000
**Nanog**	Cell Signaling technology	4903	1:1000 for WB, 1:200 for IF
**Ki-67**	ABclonal	A26200	1:500
**PCNA**	ABclonal	A12427	1:1000
**LC3B**	Cell Signaling technology	2775	1:1000
**LC3B**	Cell Signaling technology	83506	1:400 for IF
**ATG3**	Cell Signaling technology	3415	1:1000
**ATG5**	ABclonal	A19677	1:1000
**BHMT**	ABclonal	A5134	1:1000
**YTHDF1**	Cell Signaling technology	57530	1:1000
**m^6A**	ABclonal	A19841	1:1000
**ACTB**	ABclonal	AC038	1:10000
**TRITC-conjugated AffiniPure Goat Anti-Mouse IgG (H+L)**	Jackson ImmunoResearch	115-025-062	1:100
**FITC AffiniPure™ Goat Anti-Rabbit IgG (H+L)**	Jackson ImmunoResearch	111-095-003	1:100
**HRP Goat Anti-Rabbit IgG**	ABclonal	AS014	1:5000

Table S6. The primer sequences

Gene	Primers（5’→3’）
**CD133**	Fwd AGTCGGAAACTGGCAGATAGC
	Rev GGTAGTGTTGTACTGGGCCAAT
**CD44**	Fwd CTGCCGCTTTGCAGGTGTA
	Rev CATTGTGGGCAAGGTGCTATT
**SOX2**	Fwd GCCGAGTGGAAACTTTTGTCG
	Rev GGCAGCGTGTACTTATCCTTCT
**Nanog**	Fwd AATACCTCAGCCTCCAGCAGATG
	Rev TGCGTCACACCATTGCTATTCTTC
**ATG10**	Fwd AGACCATCAAAGGACTGTTCTGA
	Rev GGGTAGATGCTCCTAGATGTGAC
**ATG12**	Fwd TAGAGCGAACACGAACCATCC
	Rev CACTGCCAAAACACTCATAGAGA
**ATG13**	Fwd AGACAGTTCGTGTTGGGACAG
	Rev AGACAGTTCGTGTTGGGACAG
**ATG14**	Fwd GCAAATCTTCGACGATCCCAT
	Rev CACACCCGTCTTTACTTCCTC
**ATG16L1**	Fwd TCTGGGACATTCGATCAGAGAG
	Rev CCTTTCTGGGTTTAAGTCCAGG
**ATG3**	Fwd ACATGGCAATGGGCTACAGG
	Rev CTGTTTGCACCGCTTATAGCA
**ATG4B**	Fwd GGTGTGGACAGATGATCTTTGC
	Rev CCAACTCCCATTTGCGCTATC
**ATG5**	Fwd GGTGTCTCTCGCAGATTCATC
	Rev TCAGTCTTCGGCTGAGGTTCT
**ATG7**	Fwd CAGTTTGCCCCTTTTAGTAGTGC
	Rev CCAGCCGATACTCGTTCAGC
**BECN1**	Fwd AGAAGCTGTTTCGTCCTGTGG
	Rev AGGTGTTTCCAACATTGGCTC
**ULK1**	Fwd CCAGAGCAACATGATGGCG
	Rev CCTTCCCGTCGTAGTGCTG
**FIP200**	Fwd ATCGAAGAGTGTGTACCTACAGT
	Rev GCAGGTGGACGATCACATAAGAT
**SQSTM1**	Fwd GACTACGACTTGTGTAGCGTC
	Rev AGTGTCCGTGTTTCACCTTCC
**18S rRNA**	Fwd GTAACCCGTTGAACCCCATT
	Rev CCATCCAATCGGTAGTAGCG

Figure S1

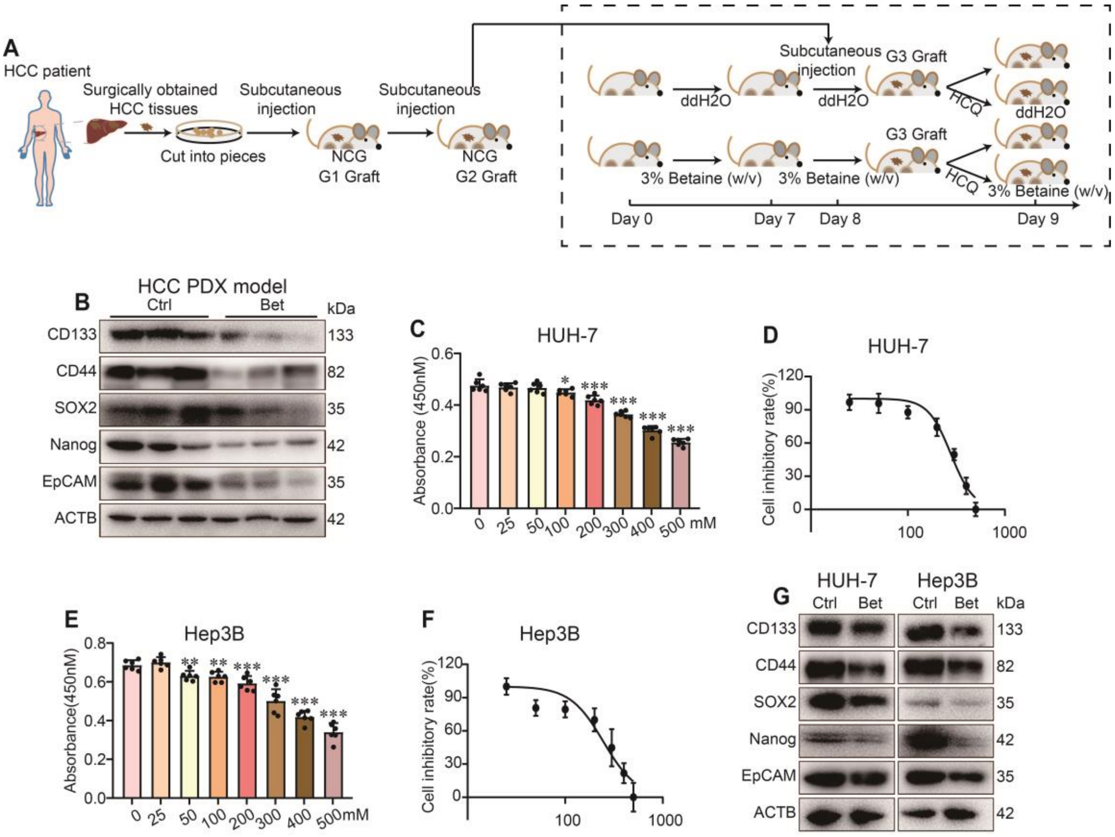

(A) Schematic diagram of the construction of the HCC PDX model and subsequent treatments. (B) The effects of betaine treatment on the expression levels of CD133, CD44, SOX2, Nanog, and EpCAM proteins in PDX tumor tissues were analyzed using WB assay. (C-F) HUH-7 and Hep3B cells were subjected to various concentrations of betaine treatment for 24 h, and cell viability was detected. The cell inhibitory rate and IC50 values of HCC cells under betaine treatment were calculated according to the absorbance. (G) HUH-7 and Hep3B cells were subjected to 200 mM of betaine treatment for 24 h, and the effects of betaine on the expression levels of CD133, CD44, SOX2, Nanog, and EpCAM in HCC cells were analyzed using WB assay. *P < 0.05, ** P < 0.01, *** P < 0.001.

Figure S2

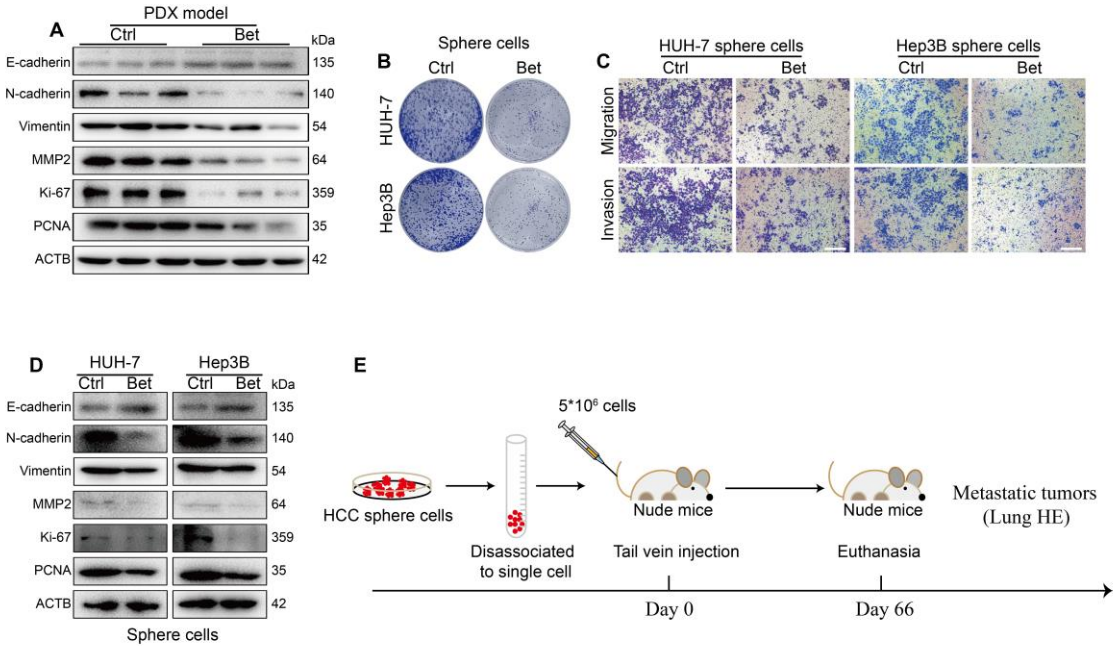

(A) The effects of betaine treatment on the expression levels of E-cadherin, N-cadherin, Vimentin, MMP2, Ki-67, and PCNA proteins in PDX tumor tissues were analyzed using WB assay. (B-C) HCC cells were treated with betaine and subjected to sphere cells formation, then sphere cells were dissociated and subjected to colony formation, and transwell migration and invasion assays. (Scale bar = 100 μm). (D) HCC cells were treated with betaine and subjected to sphere cells formation, the expression levels of E-cadherin, N-cadherin, Vimentin, MMP2, Ki-67, and PCNA proteins in HCC sphere cells were detected using WB assay. (E) Time scale diagram of tail vein-lung metastasis assay.

Figure S3

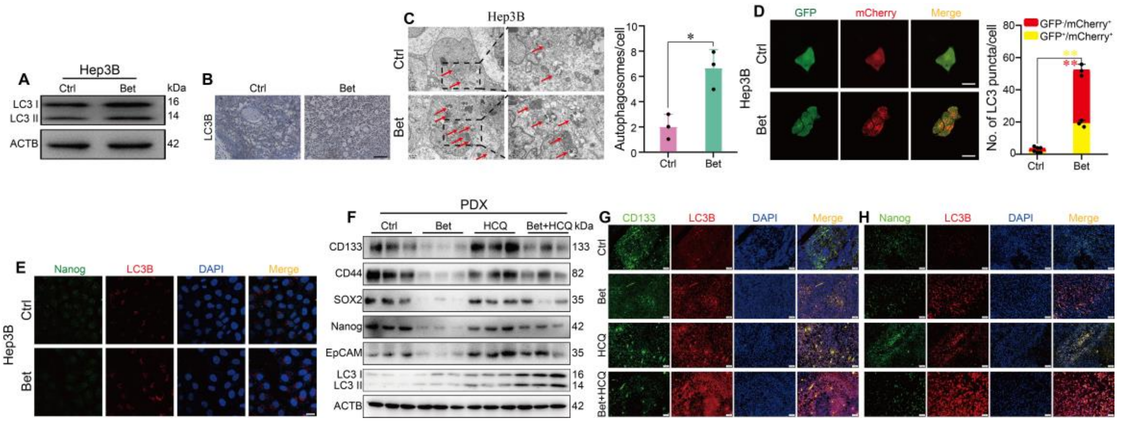

(A) Hep3B cells were treated with betaine, and the expression levels of LC3 I/II proteins were detected using WB assay. (B) After betaine treatment, the expression levels of LC3B protein in PDX

tumor tissues were detected using IHC assay (Scale bar = 100 μm). (C) Hep3B cells were treated with betaine, and the intracellular autophagosomes were captured using a TEM (scale bar = 2 μm for left panel, 1 μm for right panel). A statistical graph is shown. (D) Hep3B cells were pre-transfected with mCherry-GFP-LC3B, and then subjected to betaine treatment. The red and yellow puncta were captured by a confocal microscope and quantitated (scale bar = 20 μm). A statistical graph is shown. (E) Hep3B cells were treated with betaine, and the expression levels of Nanog and LC3B proteins in the same cells were detected using IF assay (scale bar = 20 μm). (F) After being exposed to betaine, HCQ, or combined betaine and HCQ treatments, the expression levels of CD133, CD44, SOX2, Nanog, EpCAM, and LC3 I/II proteins in PDX tumors tissues were detected using WB assay. (G-H) After being exposed to betaine, HCQ, or combined betaine and HCQ treatments, the expression levels of CD133, Nanog and LC3B in same area of PDX tumor tissues were detected via IF co-staining assay (scale bars = 50 μm). *P < 0.05, **P < 0.01, ***P < 0.001.

Figure S4

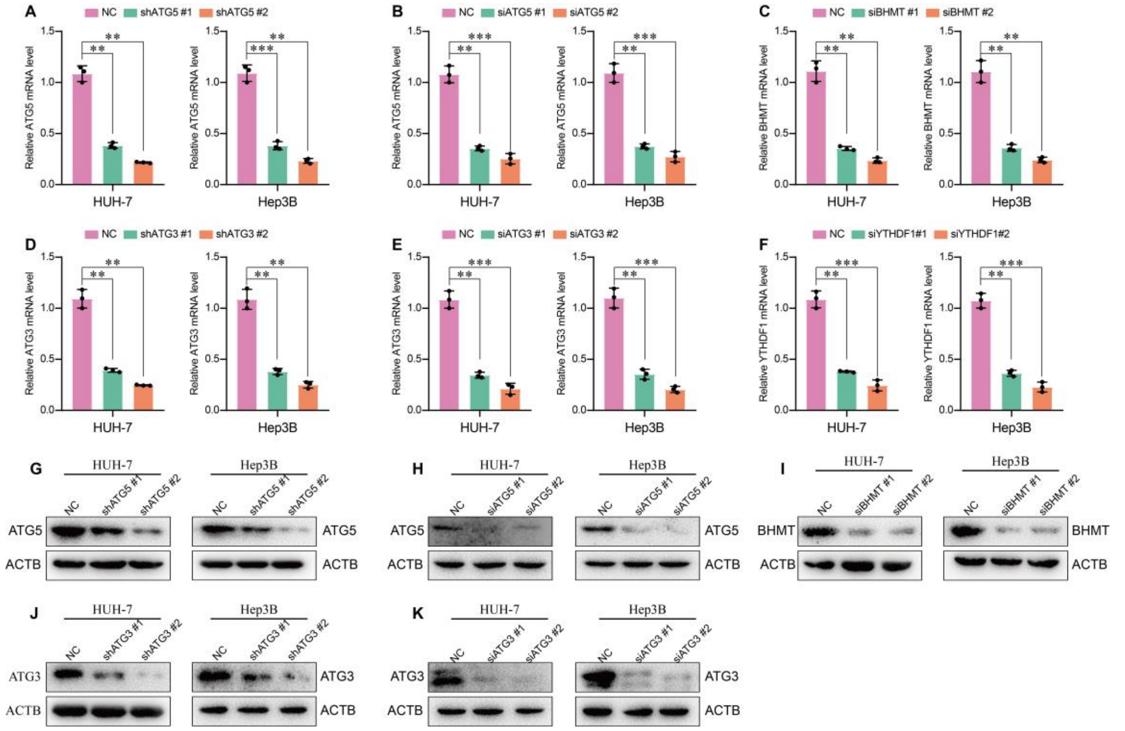

(A-F) After being transfected with shATG5, siATG5, siBHMT, shATG3, siATG3, and siYTHDF1, the expression levels of ATG5, BHMT, ATG3, and YTHDF1 mRNA in HCC cells were detected using qPCR assay. (G-K) After being transfected with shATG5, siATG5, siBHMT, shATG3, and siATG3, the expression levels of ATG5, BHMT, and ATG3 protein in HCC cells were detected using

WB assay.

Figure S5

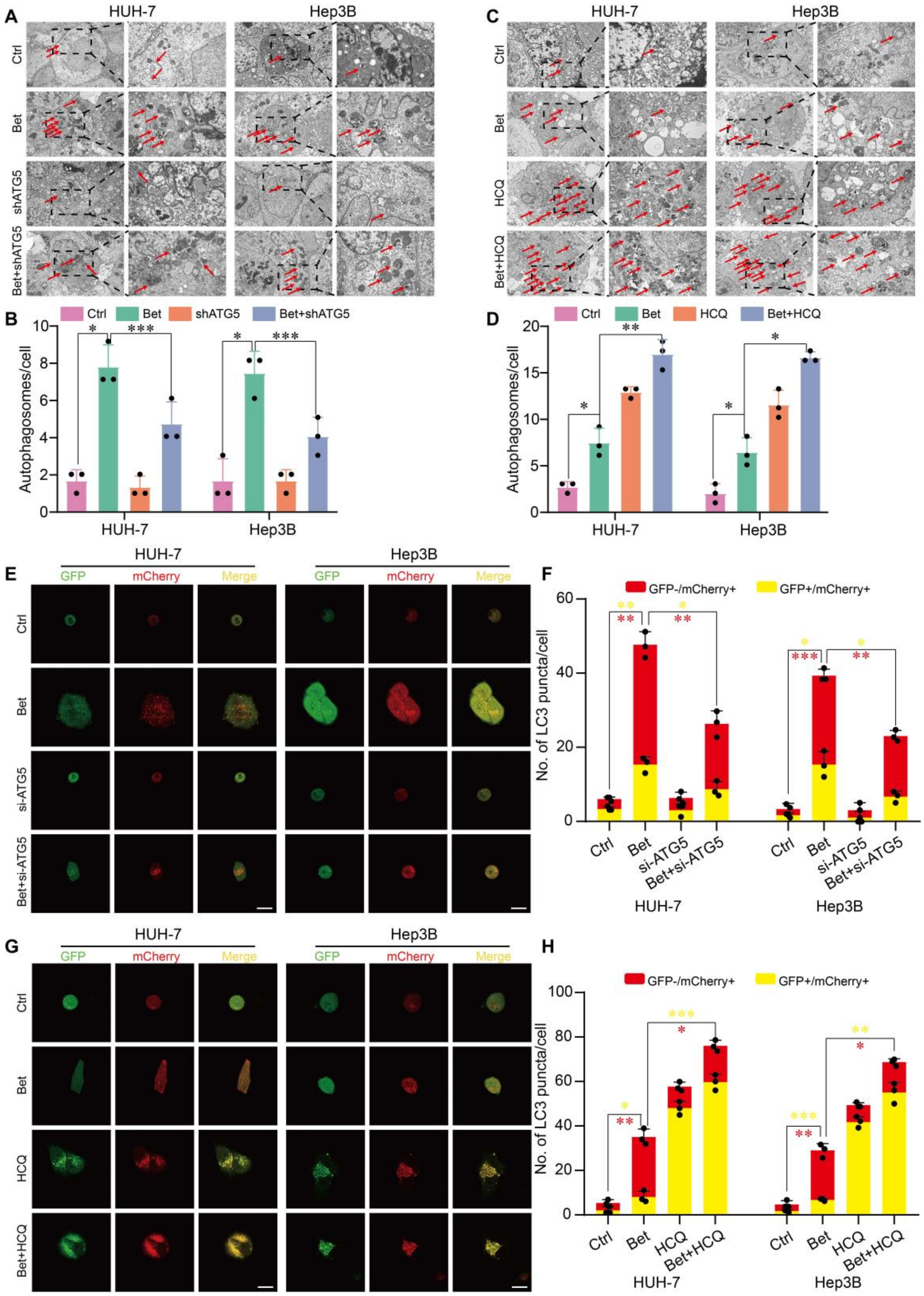

(A-B) HCC cells were pre-transfected with shATG5, and then subjected to betaine treatment. The intracellular autophagosomes were captured using a TEM (scale bar = 2 μm for left panel, 1μm for

right panel). A statistical graph is shown. (C-D) HCC cells were treated with betaine, HCQ, or combined betaine and HCQ. The intracellular autophagosomes were captured via a TEM (scale bar = 2 μm for left panel, 1μm for right panel). A statistical graph is shown. (E-F) HCC cells were pre-transfected with mCherry-GFP-LC3B and siATG5, and then subjected to betaine treatment. The red and yellow puncta were captured by a confocal microscope and quantitated (scale bar = 20 μm). A statistical graph is shown. (G-H) HCC cells were pre-transfected with mCherry-GFP-LC3B, and then subjected to betaine, HCQ, or combined betaine and HCQ treatments. The red and yellow puncta were captured by a confocal microscope and quantitated (scale bar = 20 μm). A statistical graph is shown. * $P < 0.05$, ** $P < 0.01$, *** $P < 0.001$.

Figure S6

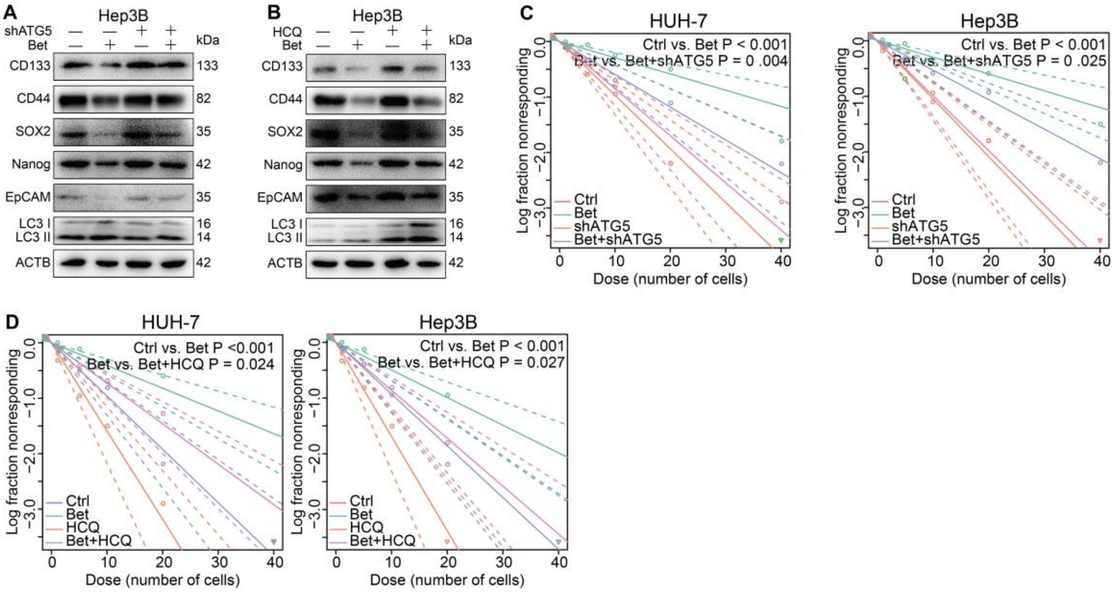

(A) Hep3B cells were pre-transfected with shATG5, and then subjected to betaine treatment. The expression levels of CD133, CD44, SOX2, Nanog, EpCAM, and LC3 I/II proteins were detected using WB assay. (B) Hep3B cells were subjected to betaine, HCQ, or combined betaine and HCQ treatments. The expression levels of CD133, CD44, SOX2, Nanog, EpCAM, and LC3 I/II proteins were detected using WB assay. (C) HCC cells were pre-transfected with shATG5, and then subjected to betaine treatment. The stemness of HCC cells under different treatments was measured using *in vitro* limiting dilution assay. (D) HCC cells were subjected to betaine, HCQ, or combined betaine and HCQ treatments, the stemness of HCC cells under different treatment was measured using *in vitro* limiting dilution assay.

Figure S7

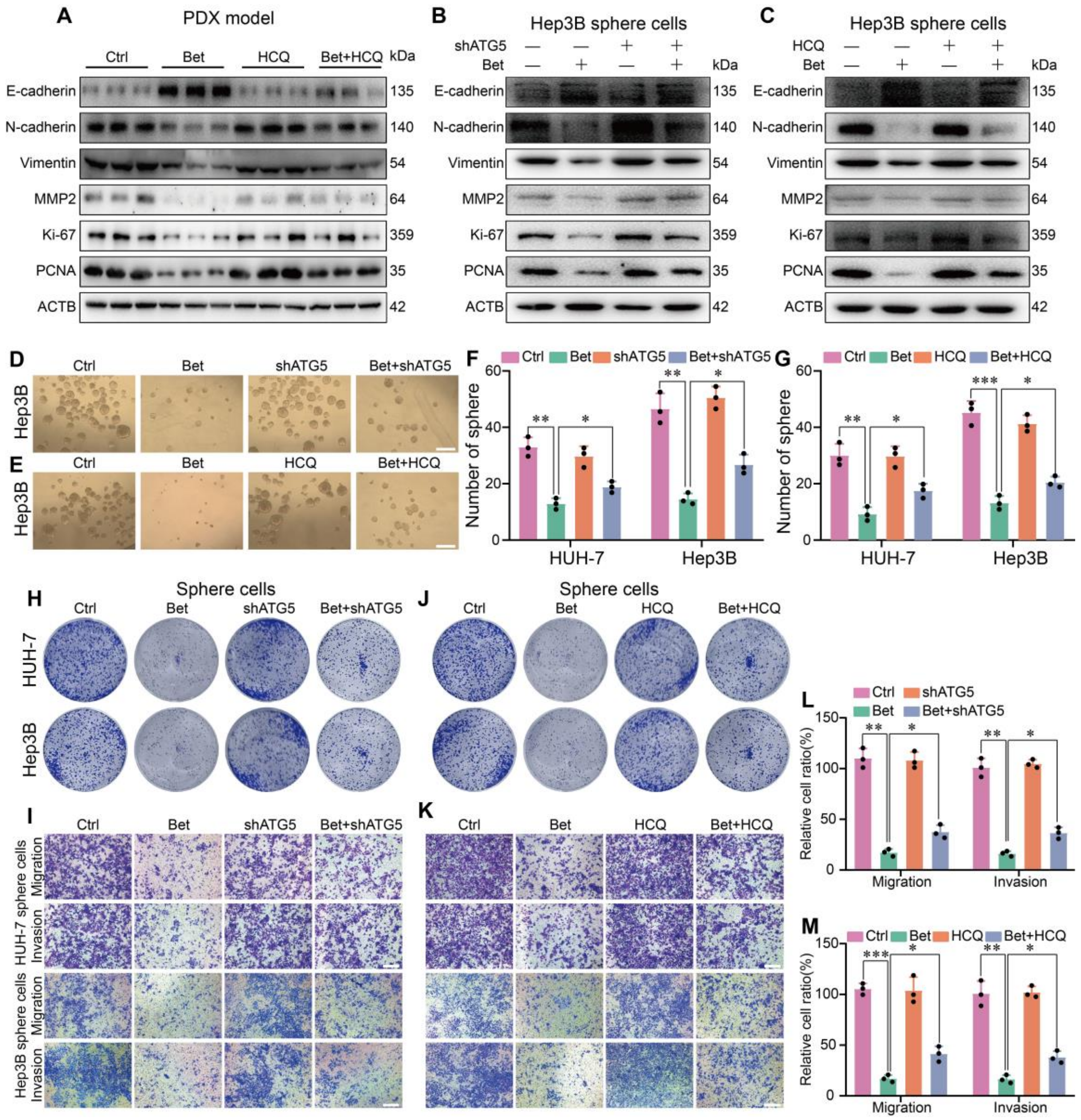

(A) After being exposed to betaine, HCQ, or combined betaine and HCQ treatments, the expression levels of E-cadherin, N-cadherin, Vimentin, MMP2, Ki-67, and PCNA proteins in PDX tumors tissues were detected using WB assay. (B) Hep3B cells were pre-transfected with shATG5 and subjected to betaine treatment for generation of sphere cells. The expression levels of E-cadherin, N-cadherin, Vimentin, MMP2, Ki-67, and PCNA proteins in Hep3B sphere cells were detected using WB assay. (C) Hep3B cells were subjected to betaine, HCQ, or combined betaine and HCQ treatments for generation of sphere cells. The expression levels of E-cadherin, N-cadherin, Vimentin, MMP2, Ki-67, and PCNA proteins in Hep3B sphere cells were detected using WB assay. (D) Hep3B cells were pre-transfected with shATG5, and then subjected to betaine treatment. The tumor sphere formation ability of HUH-7 cells under different treatments was measured (scale bars = 100 μm).

(E) Hep3B cells were subjected to betaine, HCQ, or combined betaine and HCQ treatments, and the tumor sphere formation ability of HUH-7 cells under different treatments was measured (scale bars = 100 μm). (F-G) Statistical graphs of the tumor sphere formation assays under different treatments. (H-I) HCC cells were pre-transfected with shATG5 and subjected to betaine treatment for generation of sphere cells. After being dissociated and diluted, sphere cells were subjected to colony formation, and transwell migration and invasion assays (scale bars = 100 μm). (J-K) HCC cells were subjected to betaine, HCQ, or combined betaine and HCQ treatments for generation of sphere cells. After being dissociated and diluted, sphere cells were subjected to colony formation, and transwell migration and invasion assays (scale bars = 100 μm). (L-M) Statistical graphs of transwell migration and invasion assays of Hep3B sphere cells are shown. *$P < 0.05$, **$P < 0.01$, ***$P < 0.001$.

Figure S8

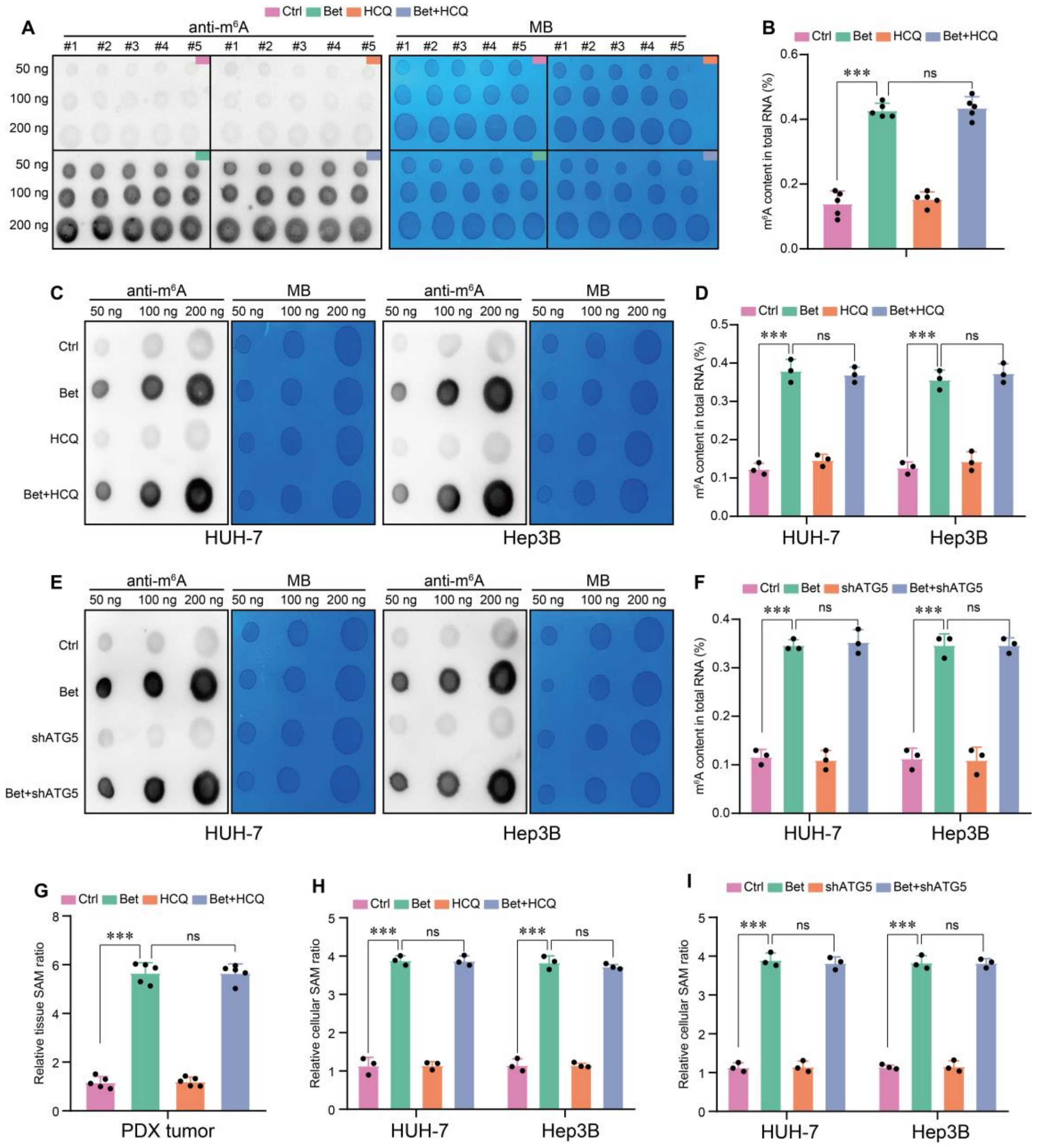

(A-B) After being exposed to betaine, HCQ, or combined betaine and HCQ, the m^{6}A modification in PDX tumor tissues were detected using dot blot and quantified using the EpiQuik m^{6}A methylation quantification kit, respectively. (C-D) After treated with betaine, HCQ or combined betaine and HCQ, the m^{6}A modification in HCC cells were detected using dot blot and quantified using the EpiQuik m^{6}A methylation quantification kit, respectively. (E-F) HCC cells were pre-transfected with shATG5, and then were subjected to betaine treatment. The m^{6}A modification in HCC cells were detected using dot blot and quantified using the EpiQuik m^{6}A methylation quantification kit, respectively. (G-H) After treated with betaine, HCQ or combined betaine and HCQ, the SAM levels in PDX tumor tissues and HCC cells were detected via SAM ELISA kit. (I)

HCC cells were pre-transfected with shATG5, and then subjected to betaine treatment. The SAM levels in HCC cells were detected via SAM ELISA kit. ns: not significant; ***P < 0.001.

Figure 9

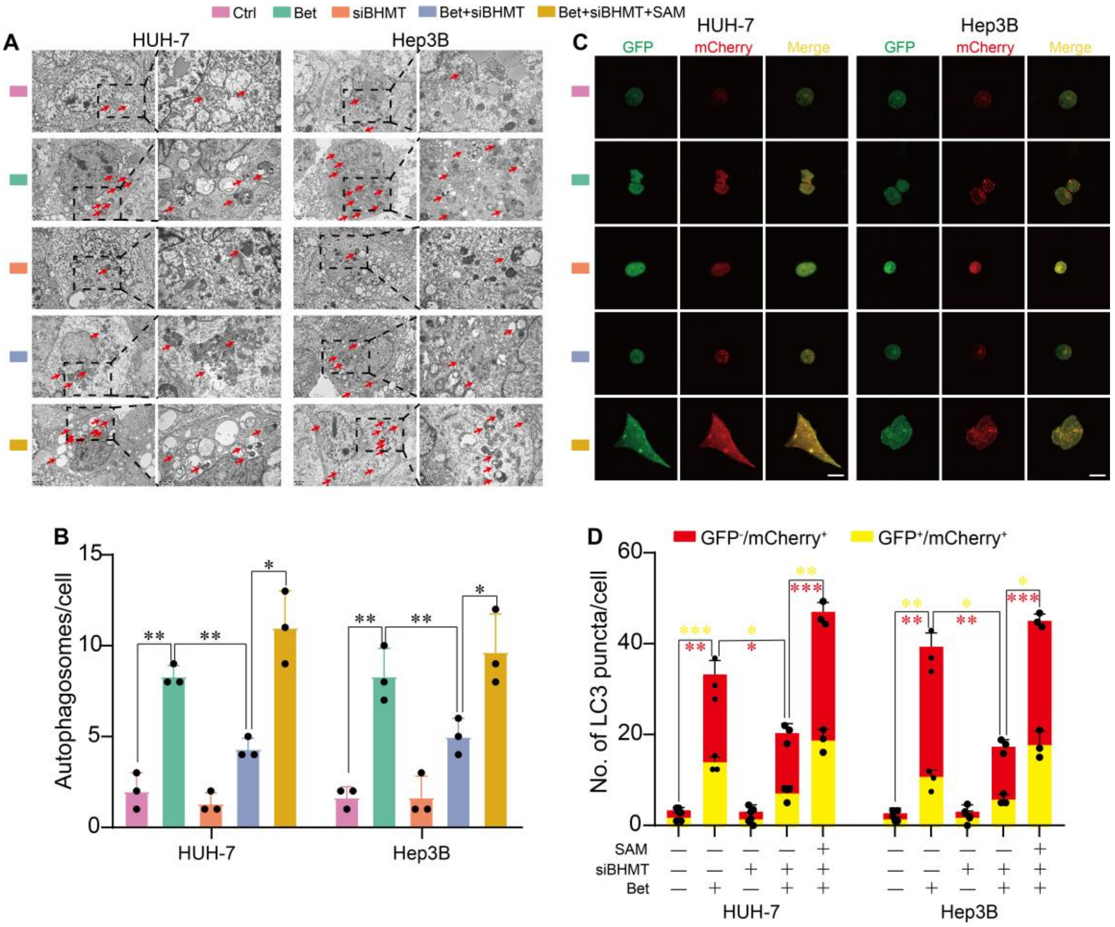

(A-B) HCC cells were pre-transfected with siBHMT, and then subjected to betaine or combined betaine and SAM treatments. The intracellular autophagosomes were captured using a TEM (scale bar = 2 μm for left panel, 1μm for right panel). A statistical graph is shown. (C-D) HCC cells were pre-transfected with mCherry-GFP-LC3B and siBHMT, and then subjected to betaine or combined betaine and SAM treatments. The red and yellow puncta were captured by a confocal microscope and quantitated (scale bar = 20 μm). A statistical graph is shown. *P < 0.05, **P < 0.01, ***P < 0.001.

Figure S10

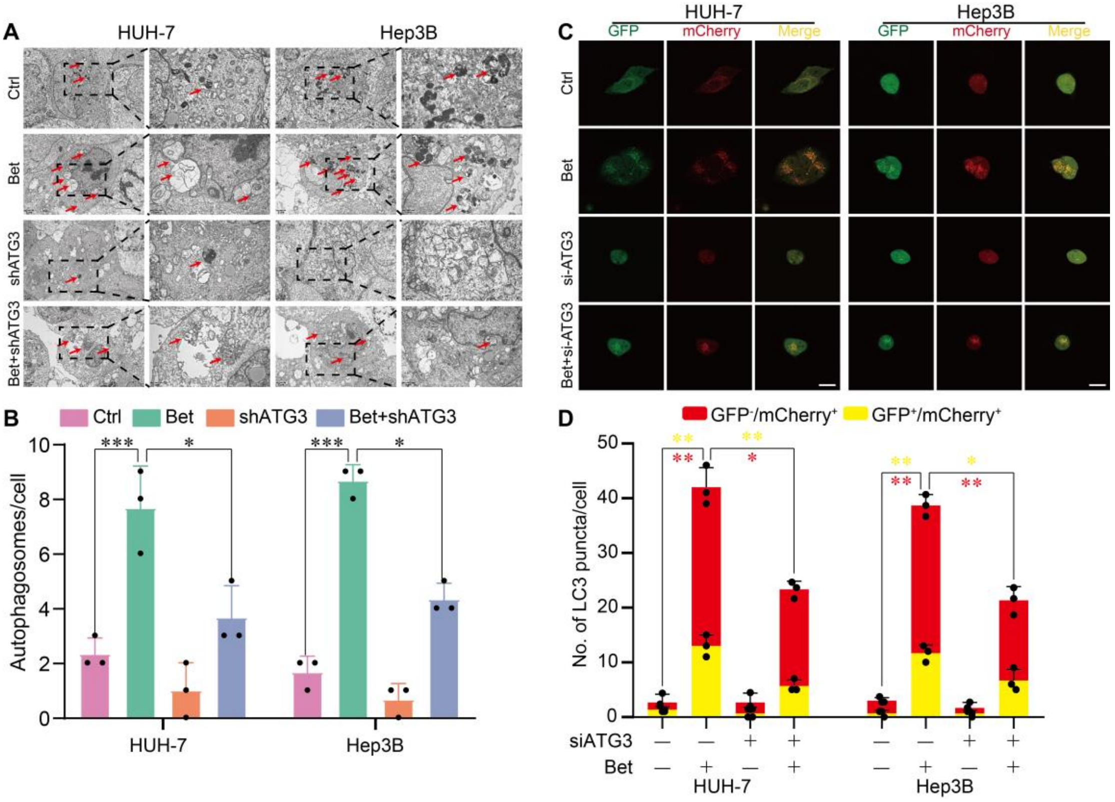

(A-B) HCC cells were pre-transfected with shATG3, and then subjected to betaine treatment. The intracellular autophagosomes were captured using a TEM (scale bar = 2 μm for left panel, 1μm for right panel). A statistical graph is shown. (C-D) HCC cells were pre-transfected with mCherry-GFP-LC3B and siATG3, and then subjected to betaine treatment. The red and yellow puncta were captured by a confocal microscope and quantitated (scale bar = 20 μm). A statistical graph is shown. *P < 0.05, **P < 0.01, ***P < 0.001.

Figure S11

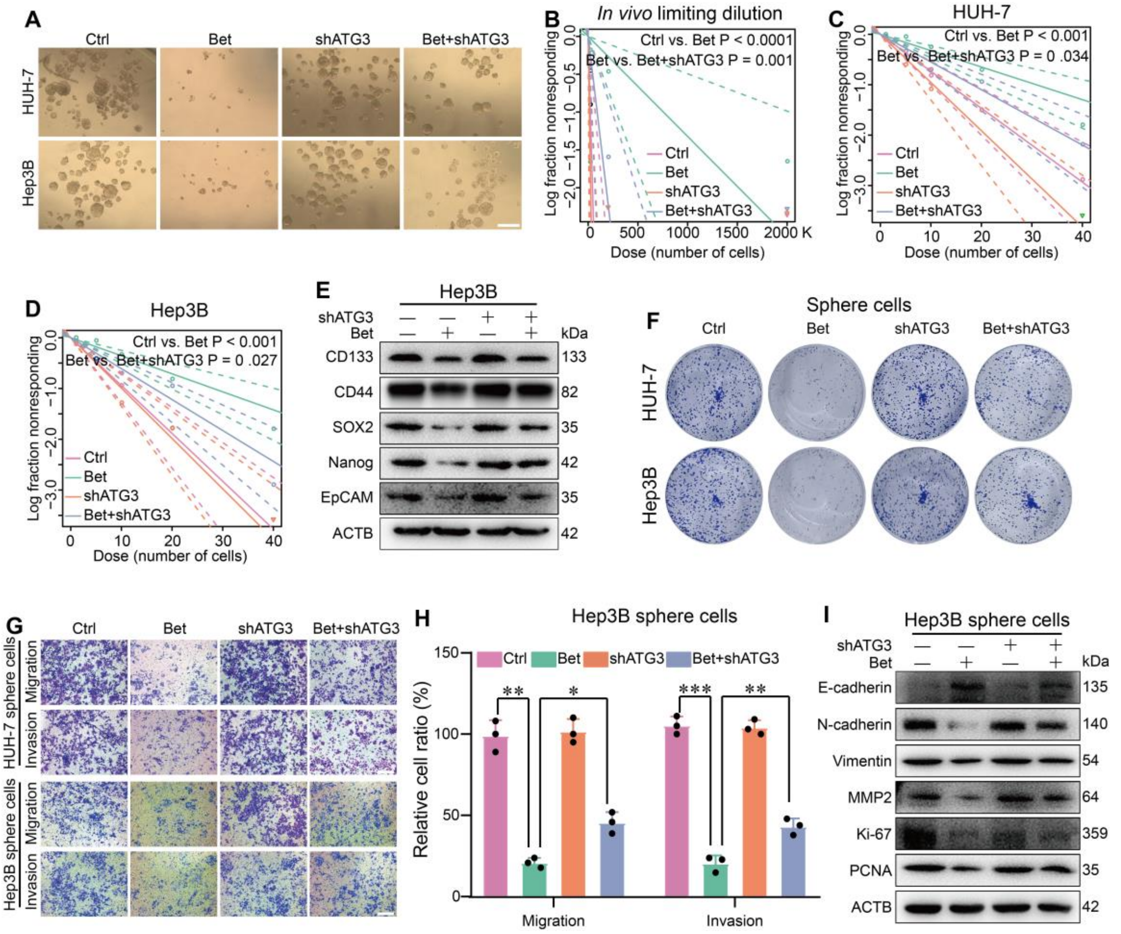

(A) HCC cells were pre-transfected with shATG3, and then treated with betaine. The tumor sphere formation ability of HCC cells under different treatments was measured (scale bars = 100 μm). (B) HUH-7 cells were pre-transfected with shATG3 and subjected to betaine treatment for generation of sphere cells. Then, HUH-7 sphere cells were diluted in a gradient and injected subcutaneously into nude mice. The number of tumors in each group was counted after 30 days. The tumor formation frequency (n = 5 per group) was shown. (C-D) HCC cells were pre-transfected with shATG3, and then subjected to betaine treatment. The stemness of HCC cells under different treatments was measured using *in vitro* limiting dilution assay. (E) Hep3B cells were pre-transfected with shATG3, and then subjected to betaine treatment. The expression levels of CD133, CD44, SOX2, Nanog, and EpCAM proteins were detected using WB assay. (F-H) HCC cells were pre-transfected with shATG3 and subjected to betaine treatment for generation of sphere cells. After being dissociated and diluted, HCC sphere cells were subjected to colony formation, and transwell migration and invasion assays (scale bar = 100 μm). A statistical graph of transwell migration and invasion assays

of Hep3B sphere cells is shown. (I) HCC cells were pre-transfected with shATG3 and subjected to betaine treatment for generation of sphere cells. The expression levels of E-cadherin, N-cadherin, Vimentin, MMP2, Ki-67, and PCNA proteins in Hep3B sphere cells were detected using WB assay. *P < 0.05, **P < 0.01, ***P < 0.001.
